# Genome-Wide Identification, Localization, and Expression Analysis of Proanthocyanidin-Associated Genes in *Brassica*

**DOI:** 10.3389/fpls.2016.01831

**Published:** 2016-12-09

**Authors:** Xianjun Liu, Ying Lu, Mingli Yan, Donghong Sun, Xuefang Hu, Shuyan Liu, Sheyuan Chen, Chunyun Guan, Zhongsong Liu

**Affiliations:** ^1^Oilseed Crops Institute, Hunan Agricultural UniversityChangsha, Hunan, China; ^2^College of Life Sciences, Resources and Environment Sciences, Yichun UniversityYichun, China; ^3^School of Biology, Hunan University of Science and TechnologyXiangtan, China

**Keywords:** *Brassica* spp., proanthocyanidin biosynthesis, gene cloning, BAC library, seed color

## Abstract

Proanthocyanidins (PA) is a type of prominent flavonoid compound deposited in seed coats which controls the pigmentation in all *Brassica* species. Annotation of *Brassica juncea* genome survey sequences showed 72 PA genes; however, a functional description of these genes, especially how their interactions regulate seed pigmentation, remains elusive. In the present study, we designed 19 primer pairs to screen a bacterial artificial chromosome (BAC) library of *B. juncea*. A total of 284 BAC clones were identified and sequenced. Alignment of the sequences confirmed that 55 genes were cloned, with every *Arabidopsis* PA gene having 2–7 homologs in *B. juncea*. BLAST analysis using the recently released *B. rapa* or *B. napus* genome database identified 31 and 58 homologous genes, respectively. Mapping and phylogenetic analysis indicated that 30 *B. juncea* PA genes are located in the A-genome chromosomes except A04, whereas the remaining 25 genes are mapped to the B-genome chromosomes except B05 and B07. RNA-seq data and Fragments Per Kilobase of a transcript per Million mapped reads (FPKM) analysis showed that most of the PA genes were expressed in the seed coat of *B. juncea* and *B. napus*, and that *BjuTT3, BjuTT18, BjuANR, BjuTT4-2, BjuTT4-3, BjuTT19-1*, and *BjuTT19-3* are transcriptionally regulated, and not expressed or downregulated in yellow-seeded testa. Importantly, our study facilitates in better understanding of the molecular mechanism underlying *Brassica* PA profiles and accumulation, as well as in further characterization of PA genes.

## Introduction

In oilseed brassicas, a yellow-seeded form is preferred over a black- or brown-seeded counterpart mainly because of a thinner seed coat and higher oil content (Friedt and Snowdon, 2009; Velasco and Ferna'ndez-Martı'nez, 2009). Importantly, proanthocyanidins (PAs) play a critical role in this differential pigmentation process (Auger et al., 2010; Fang et al., 2012; Lu et al., 2012).

Proanthocyanidins (PAs) are end-products of a well-studied branch of the flavonoid biosynthetic pathway in higher plants (Winkel-Shirley, 2001; Lepiniec et al., 2006; Saito et al., 2013). In *Arabidopsis*, a close relative of the *Brassica* species, 19 single-copy genes have been associated with PA (Appelhagen et al., 2014, 2015; Ichino et al., 2014). These genes can be divided into three classes based on their functions: structural, transcriptionally regulatory, or genes responsible for PA modification, transport, and oxidation. PA genes have also been cloned from a dozen other plant species (Hichri et al., 2011; Falcone Ferreyra et al., 2012) such as maize, and soybean (Yang et al., 2010; Senda et al., 2012). In contrast to single-copy genes in *Arabidopsis*, several plant species have multiple homologs for a given PA gene. For example, there are nine *CHS* homologs in soybean (Yi et al., 2010).

In *Brassica* species homologous cloning is used to isolate PA genes by such as *DFR*/*TT3* (Yan et al., 2008; Akhov et al., 2009), *ANS/TT18* (Yan et al., 2011), *ANR/BAN* (Nesi et al., 2009), *TT10* (Zhang et al., 2013), *TT2* (Wei et al., 2007), *TT8* (Padmaja et al., 2014), *TT12* (Chai et al., 2009), *TT16* (Deng et al., 2012; Chen et al., 2013), *TTG1* (Zhang et al., 2009; Yan et al., 2014) and *TTG2* (Li et al., 2015). However, homologous cloning has drawbacks. It needs prior knowledge of sequences of homologous gene, and is slow and difficult to amplify all members of a gene family, particularly in polyploid species, e.g., *Brassica juncea*, an allotetraploid species. To address these limitations, next-generation sequencing has been widely adopted. Up to date the genomes of over 100 plant species, including *B. rapa* (Wang et al., 2011), *B. olearcea* (Liu et al., 2014), and *B. napus* (Chalhoub et al., 2014) have been sequenced. Very recently, the genome sequence of *B. nigra* has also been released (http://www.ncbi.nlm.nih.gov/genome/10988). Whole-genome sequence annotation facilitates in genome-wide identification of PA genes (Velasco et al., 2007; Guo et al., 2014). However, the PA genes of *Brassica* species have not been analyzed in great detail. Furthermore, the complete genome sequencing of *Brassica juncea* has not been achieved to date. Yang et al. (2014) has conducted a survey of genome sequences in *B. juncea*. Genome survey sequencing (GSS) can provide information about gene content, functional elements and molecular markers (Jiao et al., 2012; Hirakawa et al., 2015), as well as compare genes of related species for the phylogenetic reconstruction of other non-model species.

Reverse transcription-polymerase chain reaction (RT-PCR), real-time fluorescent quantitative PCR, and transcriptome sequencing (RNA-seq) can analyze the spatial and temporal expression pattern, functions and interactions among various genes (Agarwal et al., 2014). RNA-seq is widely used to estimate transcript amounts and to obtain a quantitative account of transcript amounts in organisms, organs, tissues, or specific cell types, frequently comparing transcript amounts among different samples (Martin et al., 2013; Weber, 2015).

In the present study, GSS was conducted on the inbred line of *B. juncea* var. Purple-leaf Mustard (PM), and a total of 69,193 coding genes, including 72 PA genes, were predicted by annotation of GSS. Approximately 19 primer pairs specific for PA genes were then designed to screen a bacterial artificial chromosome (BAC) library of *B. juncea*, which was constructed from the same inbred line. In total, 284 BAC clones were identified and 55 *B. juncea* PA genes were confirmed by sequencing of fragments amplified from representative BAC clones. Its genomic or chromosomal positions were predicted by mapping to the sequenced *B. rapa, B. nigra*, or *B. napus* genomes, which was used as reference genomes to perform phylogenetic analysis on the full-length gene sequences and the end sequences of gene-carrying BACs. The expression level of PA genes were estimated in the seed coat and compared between the yellow- and brown-seed coat by fragments per kilobase of exon model per million mapped reads (FPKM) analysis of RNA-seq data in *B. juncea* and *B. napus*. Identification, mapping, and expression analysis of the PA genes in the present study may facilitate in better understanding the genetic mechanism underlying proanthocyanidin biosynthesis, profile, and accumulation in various *Brassica* species.

## Materials and methods

### Plant accessions

The inbred line of *B. juncea* var. PM was used for GSS and construction of the BAC library. RNA was extracted from the seed coat of the inbred line of *B. juncea* var. Sichuan Yellow (SY, yellow-seeded) and its brown-seeded near-isogenic lines (NILA and NILB), the black-seeded *B. napus* cv. Xiangyou 15 and two of its F_7_ recombinant inbred linesRIL52 and RIL55 15 days after pollination (DAP, torpedo to late torpedo stage) (Liu et al., 2009; Nesi et al., 2009). The plants were grown in a greenhouse under a photoperiod of 16 h/8 h (day/night cycle) at 22°C.

### Genome sequencing, sequence assembly, gene prediction, and annotation

Paired-end (PE) libraries were prepared using total DNA from PM, which were then constructed according to the instructions provided by Illumina (San Diego, CA, USA) with a 500-bp insert size and 125-bp read length. Sequence analyses were conducted using the Illumina HiSeq 2000 platform.

The obtained reads were subjected to quality control as follows: bases with quality scores <10 were filtered out by FastQC-0.11.3 (Schmieder and Edwards, 2011). Adaptor sequences in the reads were trimmed using fastx clipper of the FASTX-Toolkit 0.0.13 (http://hannonlab.cshl.edu/fastx_toolkit). After trimming, reads including *N* nucleotide lengths of <100 bases were excluded, and the remaining high-quality data was used for *de novo* sequence assembly by SOAP (Schmieder and Edwards, 2011). Protein-encoding sequences in the assembled genomic sequences of PM were predicted by Augustus 2.7 (Stanke and Waack, 2003) using the *A. thaliana* training set under the default parameters. Reciprocal best-hit analysis (Moreno-Hagelsieb and Latimer, 2008) was performed to compare the results of the prediction by using *B. rapa* training sets.

### Construction, pooling, and screening of the BAC library

The *B. juncea* BAC library named ZBjuH was constructed from the inbred line of the PM that were treated with the restriction endonuclease *Hind*III (Luo and Wing, 2003). This library consists of 71,808 clones with an average insert size of 126 kb genomic DNA, and an estimated 10.8-fold coverage of the *B. juncea* genome. The clones were arranged in 187 384-well plates. The clones were organized into three-dimensional BAC pools of plates, rows, and columns. The superplate consisted of 19 DNA samples, each representing 10 BAC plates, except for superplate 19, which only consists of 7 384-well plates. The first dimension consisted of the BAC clone plate of 187 DNA samples. The second and third dimensions consisted of 8 and 12 DNA samples, respectively, for the pooled 16 rows and 24 columns of the BAC clones. Screening of single BAC clones was performed in a five-step PCR process (Figure S1). The PCR primers were designed according to the conserved sequences of the PA genes that were annotated from the *B. juncea* GSS (Table S1). PCR reactions were performed in a total volume of 10 μL with a reaction mixture as follows: 10 × PCR buffer (1.0 μL), dNTP mix (10 mM each, 0.15 μL), 1 U Taq DNA polymerase (Takara, Japan), 1 μL template, 10 mM forward primer (0.5 μL), 10 mM reverse primer (0.5 μL) and ddH_2_O up to 10 μL. A “touchdown” PCR amplification program is used as follows: 94°C for 5 min; 6 cycles of 30 s at 94°C, 40 s at 62°C with a 1°C decrease in the annealing temperature per cycle, and 1 min at 72°C; 30 cycles of 30 s at 94°C, 45 s at 56°C, and 1 min at 72°C; and a final extension at 72°C for 10 min. The PCR products were observed by electrophoresis on 1.5% agarose gels using ethidium bromide and UV visualization. The BAC clones from which the fragment of expected size was amplified were considered positive BAC clones.

### Grouping and sequencing for full-length gene of positive BAC clones

Gene fragments amplified from the positive BAC clones were sequenced and aligned with annotated PA genes using DNAMAN4.0 (LynnonBiosoft, USA) to confirm whether the cloned and the annotated gene were the same copy. When a cloned gene harbored a single nucleotide difference (SNP) and/or insertion or deletion (Indels) in its sequence from the corresponding annotated gene, the cloned and the annotated genes are considered different. For each PA gene, one or two BAC clones were selected for sequencing of the full-length genes by the high-quality, longer read Sanger method (Life Technologies, Shanghai).

### Identification and phylogenetic analysis of PA genes in *B. napus, B. nigra*, and *B. rapa*

The sequences of cloned *B. juncea* PA genes were mapped to the released *B. napus* (http://www.genoscope.cns.fr/blat-server/cgi-bin/colza/webBlat), *B. nigra* (http://www.ncbi.nlm.nih.gov/genome/10988), or *B. rapa* (http://brassicadb.org/brad/blastPage.php) reference genome to search for homologous *B. napus, B. nigra* or *B. rapa* PA genes with an identity ≥90%. Phylogenetic analysis of homologous PA genes in *B. juncea, B. rapa, B. napus*, and *Arabidopsis* was performed by using neighbor-joining (NJ) method as provided in MEGA 5.2 (Tamura et al., 2011), and the reliability of the phylogenetic trees was evaluated by the bootstrap method, with 1000 replications. The *B. juncea* PA genes on the same branch (clade) of the phylogenetic tree were classified into a homologous group.

### Sequencing and mapping of BAC ends

The BACs used for full-length sequencing of the gene were also sequenced for end-sequencing on an ABI 3730X DNA analyzer (Life Technologies, Shanghai). The sequencing primers were modified pIndigoBAC536 cloning vector-derived sequencing primers M13R (5′-CAGGAAACAGCTAT-GACC-3′) and S2 (5′-CGAATTCGAGCTCGGTACCC-3′). The sequence obtained by using the primer M13R was designated as left end (L) of the BAC clone, whereas the sequence by S2 was considered the right end (R). BAC end-sequences (BESs) were also mapped to the recently sequenced *B. napus* (http://www.genoscope.cns.fr/blat-server/cgi-bin/colza), *B. nigra* (http://www.ncbi.nlm.nih.gov/genome/10988) or *B. rapa* (http://brassicadb.org) reference genome to assign a genomic location when at least 100 bp aligned to the reference genome, with at least 75% identity. If hits were obtained at multiple locations in any one of the reference genomes, then a BES was assigned to the position of the hit with the highest identity. The position of a BES was indicated by the first and the last assigned nucleotide (nt) on each reference genome.

### Expression analysis of PA genes in seed coat

Isolation, reverse transcription and RNA-seq analysis of RNA from fresh seed coats were performed as described by Liu et al. (2013). The expression level of every PA gene in the seed coat was calculated using the FPKM method (Mortazavi et al., 2008). To compare transcript abundance of cloned PA genes in seed coat between the yellow-seeded inbred SY and its brown-seeded near-isogenic lines (NILA and NILB), the respective mapped reads from the SY/NILA and the SY/NILB pairs for each gene were counted using TopHat v2.0.9 (Kim et al., 2013). Fold changes for each gene between NILs and SY were computed as the ratio of the FPKM values. When the FPKM value of NILs or SY was 0, the substitute 0.001 was used for estimation of fold change. To display changes of PA gene expression in seed coat, the heatmap was constructed by using Heml software (“Normalization:” Logarithmic Base 2, “DEMO:” Canvas) (Deng et al., 2014).

The primers used in RT-PCR expression analysis are listed in Table S2. The following cycling parameters were used for amplification of the PA genes: 1 cycle of 4 min at 94°C; 38 cycles of 50 s at 94°C, 50 s at 58°C, 1 min at 72°C; one cycle of 6 min at 72°C. The PCR products were verified by gel electrophoresis as earlier described.

## Results

### Identification and cloning of PA genes in *B. juncea*

A total of 56.2 Gb high-quality sequencing data were assembled into 835 Mb of genomic sequence, with contig and scaffold N50 sizes of 2584 bp and 16,777 bp in *B. juncea* (Table S3). A total of 233,309 coding genes were predicted by Augustus 2.7 (Table S3) and annotated by alignment of the deduced amino acid sequence to *B. rapa* genes (http://brassicadb.org/brad/). Approximately 69,193 records were screened out, with sequence identity greater than 70% and alignment length greater than 100 amino acids, which correspond to 32,798 *B. rapa* genes (Table S4). For a *B. rapa* gene, an averaged 2.1 homologs, at most 11 homologs, were detected in the *B. juncea* genome. Among the 69,193 predicted *B. juncea* genes, 72 were identified as PA genes (Table S5). The number of *B. juncea* genes that were homologous to a given *Arabidopsis* PA gene varied from two (*DFR, TT1, TT2, TT8, TTG1*, and *TT12*) to six (*TT4, TT6*, and *ANR*) (Table S5). Furthermore, two annotated *B. juncea* genes of *TT6* and *TT7* were located within the same scaffold (Table S5).

A total of 284 positive BAC clones were identified using 19 PA gene-specific primer pairs from ZBjuH BAC library (Table 1). The amplified fragments were sequenced, and 284 clean sequences with sizes between 192 and 1487 bp were obtained. Alignment showed that these fragments represented 55 *B. juncea* PA genes, corresponding to 16 *Arabidopsis* PA genes, with each *Arabidopsis* PA gene having 2–7 *B. juncea* homologs (Table 1). All cloned *B. juncea* PA genes, except for *BjuTT4-2, BjuTT4-7*, and *BjuTT16-6*, showed genomic sequences that were similar to the corresponding predicted PA genes. These amplified sequences were not evenly distributed among genes. For 6 genes, only one sequence was each identified, whereas at least 10 sequences were detected for 7 other genes. The remaining 42 genes were each carried by 2–9 BAC clones (Table 1), which is consistent with coverage of the genome by the BAC library used. No BAC clones were identified for six the annotated genes (TT4_g135394, TT5_g158015, ANR_g228640, ANR_g226654, TT19_g144296, and TT19_g167454) (Figure S3).

**Table 1:** **Grouping of the PA gene carrier BAC clones screened by PCR from ***Brassica juncea*****.

**Gene Type**	***Arabidopsis* homolog**	**Primer pair used**	**Predicted gene**	**Cloned gene**	**No. BACs**	**BAC clone(s) carrying the gene**
Structural	*TT4/CHS*	STT4	g125911	*BjuTT4-1*	5	ZBjuH038D07, ZBjuH052L16, **ZBjuH187G14**, ZBjuH187G15, ZBjuH187H15
			–	*BjuTT4-2*	14	ZBjuH036L22, ZBjuH062E10, ZBjuH068P04, ZBjuH090C04, ZBjuH103N17, ZBjuH115L06, ZBjuH117A13, ZBjuH125A20, ZBjuH130C13, ZBjuH167H05, ZBjuH167H11, ZBjuH167H12, **ZBjuH175I06**, ZBjuH187A11
			g94262	*BjuTT4-3*	16	**ZBjuH037O10**, ZBjuH040M24, ZBjuH042E09, ZBjuH054M24, ZBjuH058B18, ZBjuH102A19, ZBjuH103N21, ZBjuH110J17, ZBjuH111B11, ZBjuH119C13, ZBjuH124I11, ZBjuH129K08, ZBjuH139O13, ZBjuH143K02, ZBjuH162N03, ZBjuH165A05
			g160192	*BjuTT4-4*	5	ZBjuH031A21, ZBjuH031B12, **ZBjuH036O12**, ZBjuH048I18, ZBjuH095E01
			g112186	*BjuTT4-5*	2	ZBjuH044O21, **ZBjuH053C09**
			g134422	*BjuTT4-6*	2	**ZBjuH053C08**, ZBjuH115L05
			–	*BjuTT4-7*	4	ZBjuH049I15, ZBjuH049J15, **ZBjuH090K23**, ZBjuH121I20
	*TT5/CHI*	STT5	g10826	*BjuTT5-1*	1	**ZBjuH186N11**
			g147891	*BjuTT5-2*	1	**ZBjuH181K10**
			g94675	*BjuTT5-3*	10	ZBjuH027P19, ZBjuH036L22, ZBjuH041J15, ZBjuH058J20, ZBjuH066I18, ZBjuH066O10, **ZBjuH080G05**, ZBjuH156B18, ZBjuH158J20, ZBjuH177D05
			g153768	*BjuTT5-4*	5	ZBjuH096N21, **ZBjuH106O08**, ZBjuH108L09, ZBjuH119I24, ZBjuH122O18
	*TT6/F3H*	STT6	g93144	*BjuTT6-1*	7	ZBjuH020C14, ZBjuH048G02, ZBjuH048M11, **ZBjuH058K21**, ZBjuH120F22, ZBjuH165E24, ZBjuH181K08
			g230814	*BjuTT6-2*	5	ZBjuH058P02, ZBjuH059D03, ZBjuH076G06, **ZBjuH087J23**, ZBjuH144L06
			g34078	*BjuTT6-3*	1	**ZBjuH031F14**
			g58779	*BjuTT6-4*	8	**ZBjuH022O18**, ZBjuH025L04, ZBjuH047N11, ZBjuH088F14, ZBjuH095M08, ZBjuH106B12, ZBjuH132K03, ZBjuH132P11
			g51817	*BjuTT6-5*	6	ZBjuH106N13, ZBjH131P01, **ZBjuH143I07**, ZBjuH146J13, ZBjuH149K20, ZBjuH171M24
	*TT7/F3'H*	STT7	g118579	*BjuTT7-1*	9	ZBjuH012H01, ZBjuH025M21, ZBjuH045C24, ZBjuH063G22, ZBjuH095P11, ZBjuH105C09, ZBjuH156A19, **ZBjuH159L04**, ZBjuH175L17
			g105339/ g105340	*BjuTT7-2*	4	**ZBjuH080O14**, ZBjuH081G21, ZBjuH092C04, ZBjuH153O14
	*TT3/DFR*	SDFR	g119544	*BjuTT3-1*	7	**ZBjuH029J10**, ZBjuH043G11, ZBjuH118M13, ZBjuH119K03, ZBjuH157O03, ZBjuH157P04, ZBjuH184C12
			g127201	*BjuTT3-2*	3	ZBjuH134O05, ZBjuH175D09, **ZBjuH183H13**
	*TT18/ANS*	STT18	g16568	*BjuTT18-1*	4	**ZBjuH054O02**, ZBjuH091D16, ZBjuH181K13, ZBjuH187D05
			g178347	*BjuTT18-2*	3	ZBjuH020C14, ZBjuH181I12, **ZBjuH181K08**
			g86816	*BjuTT18-3*	3	**ZBjuH091K10**, ZBjuH097N14, ZBjuH178L19
			g114026	*BjuTT18-4*	5	ZBjuH054H16, ZBjuH093H16, **ZBjuH177N08**, ZBjuH182I21, ZBjuH187H15
	*ANR*	SANR	g97466	*BjuANR-1*	3	ZBjuH022P08, **ZBjuH082J01**, ZBjuH123C06
			g177273	*BjuANR-2*	2	**ZBjuH148I16**, ZBjuH165M04
			g228640	*BjuANR-3*	4	ZBjuH071P08, **ZBjuH116E04**, ZBjuH116I23, ZBjuH185I01
			g19699	*BjuANR-4*	1	**ZBjuH034P21**
	*TT10*	STT10-1	g60604	*BjuTT10-1*	6	ZBjuH003E23, BjuH033G01, ZBjuH048L18, ZBjuH057G09, **ZBjuH083G18**, ZBjuH152B03
		STT10-2	g161120	*BjuTT10-2*	9	ZBjuH006C17, ZBjuH019G11, **ZBjuH055H16**, ZBjuH107M03 ZBjuH121G03, ZBjuH126F02, ZBjuH140A18, ZBjuH140E23, ZBjuH144O05,
		STT10-1	g169945	*BjuTT10-3*	11	**ZBjuH021A16**, ZBjuH024M11, ZBjuH025E16, ZBjuH037G20 ZBjuH066O13, ZBjuH080H07, ZBjuH084L21, ZBjuH092L15, ZBjuH101B03, ZBjuH144O03, ZBju155P16
		STT10-2	g6758	*BjuTT10-4*	1	**ZBjuH176D10**
Regulatory	*TT1*	STT1	g65737	*BjuTT1-1*	4	ZBjuH021J20, ZBjuH036J21, ZBjuH157B22, **ZBjuH180A05**
			g10440	*BjuTT1-2*	3	ZBjuH097N03, **ZBjuH147E23**, ZBjuH176G24
	*TT2*	STT2	g27300	*BjuTT2-1*	2	**ZBjuH085H24**, ZBjuH137N11
			g136881	*BjuTT2-2*	7	ZBjuH028M22, **ZBjuH034J15**, ZBjuH061O14, ZBjuH068M24, ZBjuH135H01, ZBjuH149C17, ZBjuH172K23
	*TT8*	STT8-1	g113056	*BjuTT8-1*	5	**ZBjuH004L18**, ZBjuH038M05, ZBjuH068D18, ZBjuH122I23, ZBjuH173H05
		STT8-2	g109603	*BjuTT8-2*	3	**ZBjuH005J18**, ZBjuH033E04, ZBjuH036F18
	*TT16*	STT16-1	g141603	*BjuTT16-1*	2	ZBjuH051G23, **ZBjuH130K12**
		STT16-1	g157583	*BjuTT16-2*	6	ZBjuH046H18, ZBjuH070H21, ZBjuH082B14, **ZBjuH099A21**, ZBjuH153H13, ZBjuH171M11
		STT16-2	g150784	*BjuTT16-3*	2	**ZBjuH091L03**, ZBjuH163M05
		STT16-2	–	*BjuTT16-4*	4	ZBjuH057K05, ZBjuH057K06, **ZBjuH098G12**, ZBjuH160B19
		STT16-1	g231621	*BjuTT16-5*	7	ZBjuH061F23, ZBjuH064O06, ZBjuH070C13, ZBjuH094F17, **ZBjuH094N07**, ZBjuH131N02, ZBjuH135M11
		STT16-1	g170816	*BjuTT16-6*	11	ZBjuH013K01, ZBjuH030F17, ZBjuH049C21, **ZBjuH077C18**, ZBjuH077C23, ZBjuH081M09, ZBjuH093J03, ZBjuH142O22, ZBjuH144E18, ZBjuH152O04, ZBjuH171A06
	*TTG1*	STTG1	g228836	*BjuTTG1-1*	2	ZBjuH030O08, **ZBjuH130K10**
			g55489	*BjuTTG1-2*	6	**ZBjuH129A18**, ZBjuH135B10, ZBjuH140O11, ZBjuH182K06, ZBjuH185M13, ZBjuH185M14
	*TTG2*	STTG2	g112447	*BjuTTG2-1*	1	**ZBjuH088A24**
			g173809	*BjuTTG2-2*	3	**ZBjuH101A24**, ZBjuH131A11, ZBjuH170G21
			g118314	*BjuTTG2-3*	13	ZBjuH025O05, ZBjuH032N08, ZBjuH039D04,**ZBjuH063L13**, BjuH065B18, ZBjuH065I18, ZBjuH066D01, ZBjuH067B22, ZBjuH076O21, BjuH135G23, ZBjuH174G04, ZBjuH174O02, ZBjuH184O06
			g156630	*BjuTTG2-4*	5	ZBjuH028C13, **ZBjuH043G17**, ZBjuH77P08, ZBjuH147A11, ZBjuH162F23
Transporter	*TT12*	STT12	g29228	*BjuTT12-1*	4	ZBjuH046J03, **ZBjuH047J16**, ZBjuH148K24, ZBjuH148O16
			g146440	*BjuTT12-2*	3	**ZBjuH124J12**, ZBjuH125I12, ZBjuH150E09
	*TT19*	STT19	g72809	*BjuTT19-1*	13	ZBjuH006M03, ZBjuH037H06, ZBjuH061A09, ZBjuH064M21, ZBjuH066G20, ZBjuH092A06, ZBjuH093G08, **ZBjuH095N01**, ZBjuH140M06, ZBjuH161G15, ZBjuH165B07, ZBjuH172L17, ZBjuH185D12
			g159509	*BjuTT19-2*	6	ZBjuH062L17, ZBjuH120J17, ZBjuH143N08, **ZBjuH170C22**, ZBjuH179C03, ZBjuH181C15
			g118434	*BjuTT19-3*	5	ZBjuH021A20, ZBjuH070G12, **ZBjuH122M08**, ZBjuH164K02, ZBjuH168M17

*The BAC clone in bold was used to sequence full-length sequence of the gene*.

One or two BAC clones were chosen for each of the above mentioned PA gene groups of BAC clones and sequenced by walking to obtain full-length gene sequence. Alignment of the resultant full-length gene with its respective GSS sequence indicated that two predicted genes was in fact from the same gene because each of them was only a portion of the same gene (Table S6). Finally, 55 PA genes were confirmed in *B. juncea* by BAC sequencing (Table 2).

**Table 2:** **Proanthocyanidins-associated genes identified in ***B. rapa, B. juncea***, and ***B. napus*****.

***A. thaliana***	***B. rapa*^a^**	***B. juncea*^b^**	***B. napus*^c^**
**ENZYMES**			
AtTT4/CHS (AT5G13930)	Bra020688(A02)	*BjuTT4-1/ TT4_g135394*	BnaA02g30320D /BnaC02g05070D
	Bra023441(A02)	*BjuTT4-2/ BjuTT4-5*	BnaC02g38730D/ BnaCnng01290D
	Bra006224(A03)	*BjuTT4-3/ BjuTT4-6*	BnaA03g04590D/ BnaC03g06120D
	Bra008792(A10)	*BjuTT4-4/ BjuTT4-7*	BnaA10g19670D/ BnaC09g43250D
	Bra036307(A09)		BnaA09g29340D
AtTTT5/CHI (AT3G55120)	Bra017728(A03)	*BjuTT5-1*	BnaAnng08210D /BnaC07g45760D
	Bra003209(A07)	*BjuTT5-2/ TT5_g158015*	BnaA07g37900D/BnaCnng45660D
	Bra007142(A09)	*BjuTT5-3/BjuTT5-4*	BnaA09g34840D/BnaC08g26010D
AtTT6/F3H (AT3G51240)	Bra012862(A03)	*BjuTT6-1/ BjuTT6-4*	BnaA03g41250D/BnaC07g32140D
	Bra036828(A09)	*BjuTT6-2/ BjuTT6-5*	BnaA09g31780D/BnaC08g22640D
	Bra007813(A09)	*BjuTT6-3*	BnaA09g55810D
AtTT7/F3′H (AT5G07990)	Bra009312(A10)	*BjuTT7-1/BjuTT7-2*	BnaA10g23330D/BnaC09g47980D
AtTT3/DFR (AT5G42800)	Bra027457(A09)	*BjuTT3-1/ BjuTT3-2*	BnaA09g15710D/BnaC09g17150D
AtTT18/ANS (AT4G22880)	Bra013652(A01)	*BjuTT18-1/BjuTT18-3*	BnaA01g12530D/BnaC01g14310D
	Bra019350(A03)	*BjuTT18-2/BjuTT18-4*	BnaA03g45610D/BnaC07g37670D
AtANR (AT1G61720)	Bra021318(A01)	*BjuANR-1/BjuANR-2*	BnaA03g60670D/BnaC04g18950D
	Bra031403(A01)	*BjuANR-3/BjuANR-4*	BnaA01g36200D/BnaC01g29820D
AtTT10 (AT5G48100)	Bra020720(A02)	*BjuTT10-1/BjuTT10-3*	BnaAnng08030D /BnaC02g38340D
	Bra037510(A06)	*BjuTT10-2/BjuTT10-4*	BnaA06g30430D
**TRANSCRIPTIONAL FACTORS**			
AtTT1 (AT1G34790)	Bra028067(A09)	*BjuTT1-1/ BjuTT1-2*	BnaAnng02100D/ BnaC06g08390D
AtTT2 (AT5G35550)	Bra035532(A08)	*BjuTT2-1/BjuTT2-2*	BnaA08g29930D/BnaC08g07960D
AtTT8 (AT4G09820)	Bra037887(A09)	*BjuTT8-1/BjuTT8-2*	BnaA09g22810D/BnaC09g24870D
AtTT16 (AT5G23260)	Bra029365(A02)	*BjuTT16-1/ BjuTT16-5*	BnaAnng30140D/ BnaC02g41690D
	Bra013028(A03)	*BjuTT16-2/ BjuTT16-6*	BnaA03g39500D/BnaC02g42240D
	Bra026507(A09)	*BjuTT16-3/ BjuTT16-4*	BnaA09g05410D/BnaC09g04950D
AtTTG1 (AT5G24520)	Bra009770(A06)	*BjuTTG1-1/ BjuTTG1-2*	BnaC07g29950D
AtTTG2 (AT2G37260)	Bra023112(A03)	*BjuTTG2-1/ BjuTTG2-3*	BnaA03g17120D/BnaC03g20650D
	Bra005210(A05)	*BjuTTG2-2/ BjuTTG2-4*	BnaA05g07220D/BnaC04g08020D
**TRANSPORTERS**			
AtTT12 (AT3G59030)	Bra003361(A07)	*BjuTT12-1/ BjuTT12-2*	BnaA07g18120D/BnaC06g17050D
AtTT19 (AT5G17220)	Bra023602(A02)	*BjuTT19-1/BjuTT19-3*	BnaA02g03440D/BnaC02g07090D
	Bra008570(A10)	*BjuTT19-2/ TT19_g144296*	BnaA10g17440D/BnaC09g40740D

a from http://brassicadb.org/brad/;

b this study;

c *from http://www.genoscope.cns.fr/brassicanapus/*.

### Genomic locations of PA genes in *Brassica* species

BLAST of these cloned 55 *B. juncea* PA genes against the *B. rapa* or *B. napus* reference genome identified 31 and 58 homologous genes in *B. rapa* and *B. napus*, respectively (Table 2). The neighbor-joining tree of the PA genes from *B. juncea, B. rapa, B. napus*, and *Arabidopsis* showed that *TT4* genes were clustered into five homologous groups, *TT5, TT6*, and *TT16* each into three groups; *TT10, TT18, TTG2*, and *TT19* each into two groups; and the remaining *TT3, TT7, ANR, TT1, TT2, TT8, TTG1*, and *TT12* genes were clustered into only one homologous group, indicating that these genes were highly conserved in terms of genomic sequence (Figure S2).

**Figure 1:**
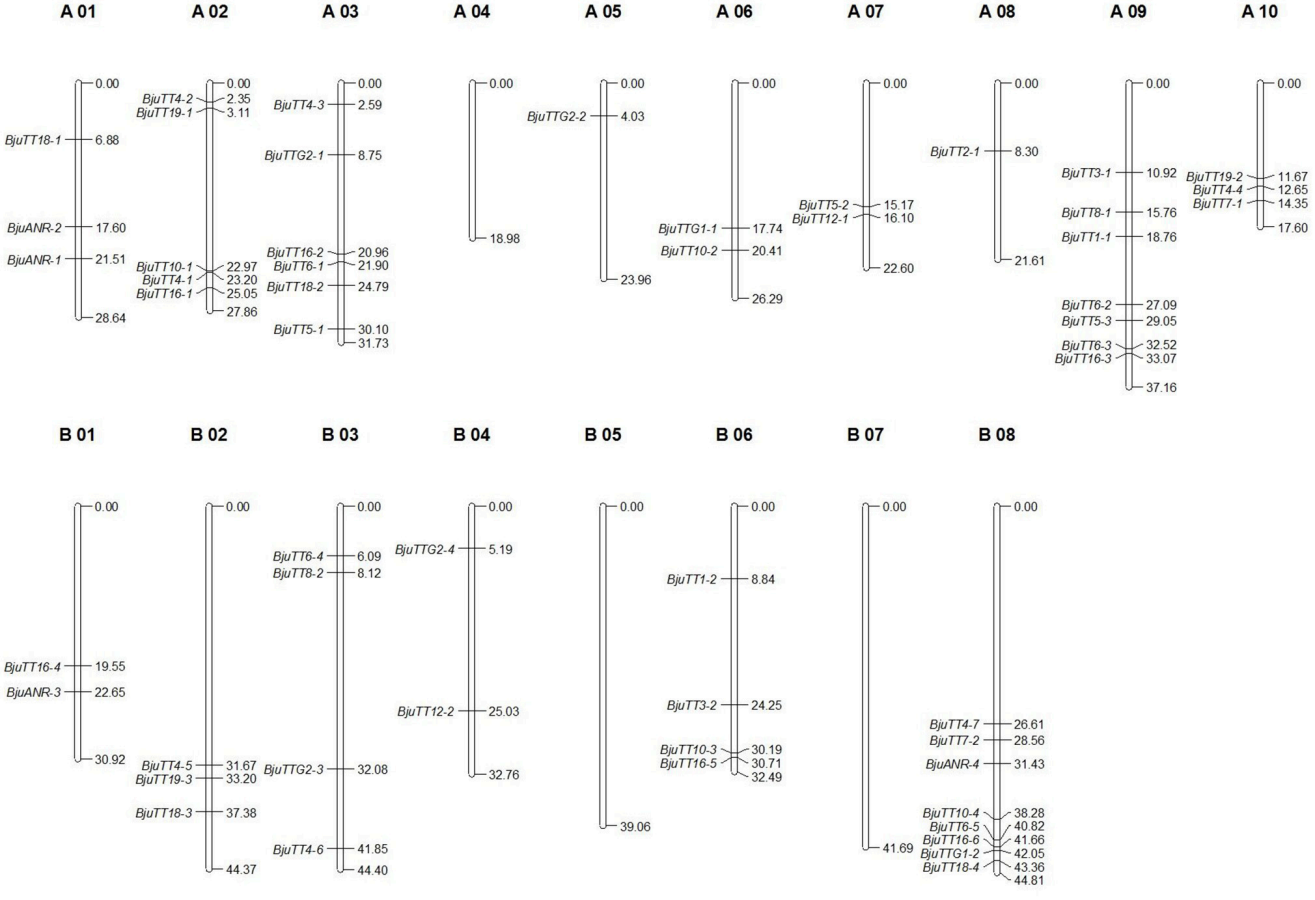
**Putative chromosomal positions of cloned proanthocyanidins-associated genes in ***Brassica juncea*****. *BjuTT5-4* and *BjuTT2-2* were not located on the chromosome in *B. juncea*.

Mapping of these cloned 55 *B. juncea* PA genes to the *B. rapa, B. nigra*, or *B. napus* reference genome indicated that 30 and 29 PA genes were homologous to the genes located in A-genome chromosomes except A04 of *B. rapa* and *B. napus*, respectively, whereas 23 of the other 25 genes were located in the B-genome chromosomes except B05 and B07 of *B. nigra*, the remaining two gene (*BjuTT5-4* and *BjuTT2-2*) were anchored on scaffold_30.1 and scaffold_500.1 of *B. nigra*, respectively, which have not yet been mapped onto a chromosome (Table 3, Figure 1). These PA genes have >95 identity (Table 3). Moreover, 23 of these A-genome PA genes were, respectively, located on the same chromosomes in *B. rapa* and *B. napus*, but additional genes may be located in either the same or different A-genome chromosomes or C-genome chromosomes because their positions have not been mapped to the *B. napus* reference genome (Table 3). The B-genome and the C-genome contributed 25 and 29 PA genes to *B. juncea* and *B. napus* genome, respectively, which is approximately equal to the number of PA genes from the A-genome.

**Table 3:** **Mapping to the ***Brassica rapa, B. nigra***, or ***B. napus*** reference genome of full-length sequences of the ***B. juncea*** PA genes cloned in this study**.

***B. juncea* gene**	**BAC sequenced**	**Sequence length (bp)**	**Position in *B. rapa*/*B. nigra* reference genome**	**Coverage (%)**	**Identity (%)**	**Putative genome or chromosome**	**Corresponding *B. rapa* homolog**	**Position in *B. napus* reference genome**	**Coverage (%)**	**Identity (%)**	**Putative genome or chromosome**	**Corresponding *B. napus* homolog**
*BjuTT4-1*	ZBjuH187G14	1269	A02(23204660-23205926)	98.7	98.9	A02	Bra020688	A02(21961707-21962975)	100	99.2	A02	BnaA02g30320D
*BjuTT4-2*	ZBjuH175I06	1454	A02(2357734-2359185)	98.0	97.9	A02	Bra023441	C02(2648298-2649755)	99.7	96.6	–	BnaC02g38730D
*BjuTT4-3*	ZBjuH037O10	1458	A03(2596137-2597594)	92.4	99.3	A03	Bra006224	A03(2138849-2140306)	100	99.8	A03	BnaA03g04590D
*BjuTT4-4*	ZBjuH036O12	1263	A10(12657235-12655973)	99.0	99.0	A10	Bra008792	A10(13887677-13888939)	100	99.3	A10	BnaA10g19670D
*BjuTT4-5*	ZBjuH053C09	1516	B02(31676065-31677577)	100	97.8	B02	–	C02(2648298-2649755)	96.2	93.7	–	BnaCnng01290D
*BjuTT4-6*	ZBjuH053C08	1352	B03(41854375-41855800)	94.8	96.2	B03	–	C03(2967996-2969460)	92.3	94.3	–	BnaC03g06120D
*BjuTT4-7*	ZBjuH090K23	1267	B08(26615339-26614079)	100	96.9	B08	–	A10(13887677-13888939)	99.7	93.5	–	BnaC09g43250D
*TT4-135394*		1387	B06(30337366-30338627)	97.3	94.4	B03	–	A03(2138849-2140306)	96.7	94.8	–	BnaC02g05070D
*BjuTT5-1*	ZBjuH186N11	1358	A03(30108206-30109397)	98.9	99.0	A03	Bra017728	C07(43667810-43668920)	93.2	95.5	–	BnaC07g45760D
*BjuTT5-2*	ZBjuH181K10	1526	A07(15175119-15173091)	71.1	97.1	A07	Bra003209	A07_random(1352839-1353912)	70.4	100	A07	BnaA07g37900D
*BjuTT5-3*	ZBjuH080G05	1621	A09(29057157-29055564)	98.6	97.8	A09	Bra007142	A09(25461126-25462763)	99.0	98.7	A09	BnaA09g34840D
*BjuTT5-4*	ZBjuH106O08	1625	scaffold_30.1(142586-144208)	99.7	98.8	B	–	C08(27510384-27511983)	98.5	91.9	–	BnaC08g26010D
*TT5-g158015*		1667	B04(26277974-26279709)	96.1	95.2	B04	–	Un_random(67254442-67255968)	91.6	92.4	–	BnaCnng45660D
*BjuTT6-1*	ZBjuH058K21	1343	A03(21908585-21910045)	82.4	97.0	A03	Bra012862	A03(20668741-20670084)	99.9	99.4	A03	BnaA03g41250D
*BjuTT6-2*	ZBjuH087J23	1514	A09(27095567-27097080)	100.0	100.0	A09	Bra036828	C08(25256551-25258093)	98.1	97.6	–	BnaA09g31780D
*BjuTT6-3*	ZBjuH031F14	2998	A09(32529280-32526116)	94.0	98.0	A09	Bra007813	A09_random(3426703-3429867)	94.7	99.8	A09	BnaA09g55810D
*BjuTT6-4*	ZBjuH022O18	1820	B03(6099152-6097595)	93.2	98.5	B03	–	A09(23688234-23689696)	80.4	92.7	–	BnaC07g32140D
*BjuTT6-5*	ZBjuH143I07	1454	B08(40828252-40826791)	99.1	98.1	B08	–	C07(35957629-35959030)	96.4	91.9	–	BnaC08g22640D
*BjuTT7-1*	ZBjuH159L04	2742	A10(14358845-14356094)	89.4	99.2	A10	Bra009312	A10(15436550-15439304)	99.5	98.8	A10	BnaA10g23330D
*BjuTT7-2*	ZBjuH080O14	2989	B08(28566520-28563560)	98.6	98.4	B08	–	C09(47019883-47026924)	44.4	94.1	–	BnaC09g47980D
*BjuTT3-1*	ZBjuH029J10	1556	A09(10927890-10926334)	98.3	98.3	A09	Bra027457	A09(9168455-9170011)	99.9	100	A09	BnaA09g15710D
*BjuTT3-2*	ZBjuH183H13	1689	B06(24256936-24255214)	96.1	99.5	B06	–	C09(10927890-10926334)	92.5	94.2	–	BnaC09g17150D
*BjuTT18-1*	ZBjuH054O02	1422	A01(6887113-6885692)	98.5	98.5	A01	Bra013652	A01(6294305-6295726)	100	100	A01	BnaA01g12530D
*BjuTT18-2*	ZBjuH181K08	1161	A03(24797395-24796232)	96.3	96.2	A03	Bra019350	A03(23215394-23216555)	99.9	97.3	A03	BnaA03g45610D
*BjuTT18-3*	ZBjuH091K10	1143	B02(37385323-37384165)	99.2	95.3	B02	–	C01(9585700-9587061)	83.9	93.8	–	BnaC01g14310D
*BjuTT18-4*	ZBjuH177N08	1152	B08(43361751-43360600)	98.1	95.8	B08	–	C07(39327212-39328382)	98.4	94.2	–	BnaC07g37670D
*BjuANR-1*	ZBjuH082J01	1433	A01(21514882-21513450)	99.2	99.2	A01	Bra021318	A03_random(5666520-5667956)	99.7	99.2	A03	BnaA03g60670D
*BjuANR-2*	ZBjuH148I16	1466	A01(17603658-17602193)	99.3	99.3	A01	Bra031403	A01(1630437-1631902)	100	100	A01	BnaA01g36200D
*BjuANR-3*	ZBjuH116E04	1499	B01(22657990-22659506)	98.6	96.8	B01	–	C01(28124388-28125894)	99.5	90.5	–	BnaC01g29820D
*BjuANR-4*	ZBjuH034P21	1400	B08(31434047-31435447)	99.9	99.1	B08	–	C04(18894005-18895401)	99.8	94.6	–	BnaC04g18950D
*BjuTT10-1*	ZBjuH083G18	3491	A02(22971851-22968361)	99.9	98.8	A02	Bra020720	Un_random(41316930-41322490)	62.8	94.9	–	BnaAnng08030D
*BjuTT10-2*	ZBjuH055H16	2297	A06(20410060-20412553)	91.3	99.7	A06	Bra037510	A06(20553612-20555918)	99.6	99.2	A06	BnaA06g30430D
*BjuTT10-3*	ZBjuH021A16	2838	B06(30198717-30195455)	87.0	97.4	B06	–	C02(41316930-41322490)	51.0	91.5	–	BnaC02g38340D
*BjuTT10-4*	ZBjuH176D10	2293	B08(38281497-20412553)	98.9	99.0	B08	–	A06(20553612-20555918)	99.4	93.1	–	–
*BjuTT1-1*	ZBjuH180A05	1761	A09(18767007-18765243)	99.8	97.5	A09	Bra028067	Un_random(4808305-4809953)	93.6	97.9	–	BnaAnng02100D
*BjuTT1-2*	ZBjuH147E23	1707	B06(8841257-8839558)	100	97.4	B06	–	C06(9366519-9368246)	98.8	93.2	–	BnaC06g08390D
*BjuTT2-1*	ZBjuH085H24	945	A08(8306171-8305232)	96.5	98.9	A08	Bra035532	A08_random(1033684-1034627)	100	99.5	A08	BnaA08g29930D
*BjuTT2-2*	ZBjuH034J15	944	scaffold_500.1(70364-69421)	100	100	B	–	C08(11760224-11761157)	98.9	93.3	–	BnaC08g07960D
*BjuTT8-1*	ZBjuH004L18	3551	A09(15769736-15773288)	80.2	97.7	A09	Bra037887	A09(15413735-15417282)	99.9	99.3	A09	BnaA09g22810D
*BjuTT8-2*	ZBjuH005J18	2768	B03(8122342-8125109)	100	99.7	B03	–	C09(23189158-23191902)	99.1	94.1	–	BnaC09g24870D
*BjuTT16-1*	ZBjuH130K12	1954	A02(25055704-25053743)	98.3	99.5	A02	Bra029365	Un_random(101450485-101452437)	99.9	99.6	–	BnaAnng30140D
*BjuTT16-2*	ZBjuH099A21	2258	A03(20961426-20959132)	92.9	99.4	A03	Bra013028	A03(19707165-19709160)	88.4	98.5	A03	BnaA03g39500D
*BjuTT16-3*	ZBjuH091L03	2004	A09(3307401-3305402)	45.1	97.6	A09	Bra026507	A09(2642192-2644190)	99.7	98.9	A09	BnaA09g05410D
*BjuTT16-4*	ZBjuH098G12	1981	B01(19554897-19552670)	89.0	96.9	B01		C09(2859965-2861956)	99.4	91.3	–	BnaC09g04950D
*BjuTT16-5*	ZBjuH094N07	2129	B06(30711288-30713462)	97.5	97.6	B06	–	Un_random(101450485-101452437)	91.7	92.1	–	BnaC02g41690D
*BjuTT16-6*	ZBjuH077C18	1980	B08(41662625-41664607)	99.8	98.1	B08	–	C02(44915780-44917790)	98.5	92.6	–	BnaC02g42240D
*BjuTTG1-1*	ZBjuH130K10	1582	A06(17740552-17739539)	99.7	98.8	A06	Bra009770	A06(18525005-18526105)	69.6	93.9	A06	–
*BjuTTG1-2*	ZBjuH129A18	1014	B08(42051009-42049996)	100	98.6	B08	–	C07(34623713-34624389)	66.8	92.8	–	BnaC07g29950D
*BjuTTG2-1*	ZBjuH088A24	1516	A03(8752727-8754251)	96.4	96.0	A03	Bra023112	A03(8032043-8033567)	99.4	97.5	A03	BnaA03g17120D
*BjuTTG2-2*	ZBjuH101A24	1466	A05(4037093-4035604)	96.5	97.2	A05	Bra005210	A05(3894221-3895658)	99.4	96.3	A05	BnaA05g07220D
*BjuTTG2-3*	ZBjuH063L13	1528	B03(32083362-32081848)	100	97.2	B03	–	C03(10964876-10966395)	99.2	93.1	–	BnaC03g20650D
*BjuTTG2-4*	ZBjuH043G17	1501	B04(5191336-5189901)	100	95.1	B04	–	C04(6027042-6028503)	97.4	90.9	-	BnaC04g08020D
*BjuTT12-1*	ZBjuH047J16	2487	A07(16102336-16104823)	95.1	95.5	A07	Bra003361	A07(14915288-14917797)	99.1	96.9	A07	BnaA07g18120D
*BjuTT12-2*	ZBjuH124J12	2505	B04(25039840-25037376)	98.4	92.8	B04	–	C06(19784039-19786887)	87.9	94.1	–	BnaC06g17050D
*BjuTT19-1*	ZBjuH095N01	808	A02(3117740-3118547)	98.6	98.6	A02	Bra023602	A02(1531517-1532324)	100	99.9	A02	BnaA02g03440D
*BjuTT19-2*	ZBjuH170C22	800	A10(11678470-11677671)	99.9	99.9	A10	Bra008570	A10(12914260-12915058)	99.9	99.7	A10	BnaA10g17440D
*BjuTT19-3*	ZBjuH122M08	1030	B02(33205499-33206525)	100	96.7	B02	–	C02(3777795-3778584)	85.6	95.2	–	BnaC02g07090D
*TT19-g144296*		825	B08(25314277-25313454)	100	99.4	B08	–	A10(12914260-12915058)	96.4	91.1	–	–

To confirm the above genomic locations, the BAC clones used for sequencing full-length genes were also sequenced for BESs. The resulting BESs between 587 and 1233 bp in length were also mapped in a similar way. Mapping of the BESs to the *B. rapa* reference genome showed that both BESs of 23 A-genome *B. juncea* PA genes were mapped around the genomic position as mapped by the full-length sequence of the corresponding genes. However, one BES of the BACs carrying two A-genome genes, i.e., *BjuTT2-1* and *BjuTTG1-1* was mapped to an unfixed scaffold, whereas one BES of the BACs carrying the remaining five A-genome genes, i.e., *BjuTT5-2, BjuTT6-1, BjuANR-2, BjuTT10-1*, and *BjuTTG2-1* was mapped to an unexpected genomic position (Table 4). Mapping of the BESs to the *B. napus* reference genome generated a more complicated picture. For only 15 A-genome *B. juncea* PA genes, both BESs were mapped around the genomic position as mapped by the full-length sequence of the corresponding genes. One or both BESs of the BACs carrying 7 A-genome genes, i.e., *BjuTT5-1, BjuTT6-2, BjuTT7-1, BjuTT16-2, BjuTT1-1, BjuTT2-1*, and *BjuTTG1-1* were mapped to an unfixed scaffold, whereas one or both BESs of the BACs carrying the remaining 8 A-genome genes were mapped to an unexpected A-genome chromosome, or a C-genome chromosome in *B. napus* reference genome (Table 4). Mapping of the BESs to the *B. nigra* reference genome showed that both BESs of 19 B-genome *B. juncea* PA genes were mapped around the genomic position as mapped by the full-length sequence of the corresponding genes, one BES of the BACs carrying three B-genome genes, i.e., *BjuTT4-6, BjuTT18-4*, and *BjuTT7-2* was mapped to an unexpected genomic position in the *B. nigra* reference genome, and then one BES of the BACs carrying the remaining three B-genome genes, i.e., *BjuTT5-4, BjuTT1-2*, and *BjuTT2-2* was mapped to an unfixed scaffold (Table 4).

**Table 4:** **Mapping to the ***B. rapa, B. nigra***, or ***B. napus*** reference genome of end sequences of the PA gene carrier BACs from ***B. juncea*****.

***B. juncea* gene**	**BAC sequenced**	**Left end**			**Right end**			**Putative Genome or chromosome**
		**Length (bp)**	**Position in *B. rapa*^a^, *B. napus*^b^ or *B. nigra* reference genome**	**Identity (%)**	**Length (bp)**	**Position in *B. rapa*^a^, *B. napu*^b^*s* or *B. nigra* reference genome**	**Identity (%)**	
BjuTT4-1	ZBjuH187G14	1048	A02(23296428-23295380)^a^	98.9	1113	A02(23152779-23153899)^a^	97.7	A02
			A02(22075270-22077756)^b^	99.5		A02 (21892025-21893145)^b^	99.6	
BjuTT4-2	ZBjuH175I06	1071	A02(2363318-2361982)	97.5	964	A02(2240655-2241616)	96.1	A02
			A02 (887331-888410)	99.6		A02 (755926-756886)	99.2	
BjuTT4-3	ZBjuH037O10	1080	A03(2659211-2658129)	99.0	587	A03(2525990-2526576)	99.1	A03
			A03(2210720-2211802)	100		A03(2079518-2080104)	97.5	
BjuTT4-4	ZBjuH036O12	1031	A10(12537033-12538063)	97.4	1059	A10(12660016-12659255)	97.8	A10
			A10(13765877-13766904)	98.1		A10(13890039-13890915)	98.2	
BjuTT4-5	ZBjuH053C09	1062	B02(31653057-31654113)	94.2	994	B02(31799935-31798937)	95.2	B02
BjuTT4-6	ZBjuH053C08	1110	B03(41773983-41775087)	97.5	1097	Repeat sequence	–	B03
BjuTT4-7	ZBjuH090K23	1065	B08(26731199-26730093)	93.2	1111	B08(26612733-26613845)	92.3	B08
BjuTT5-1	ZBjuH186N11	1023	A03(30133579-30132560)	98.0	1112	A03(30031062-30032157)	98.3	A03
			Un_random(22402145-22403163)	98.6		A03(28012499-28013608)	97.6	
BjuTT5-2	ZBjuH181K10	1071	A07(15073257-15074276)	97.0	926	A09(31899835-31899128)	97.4	A07
			A07(13943509-13944466)	95.7		A06(7618337-7619247)	97.5	
BjuTT5-3	ZBjuH080G05	997	A09(29107181-29106192)	99.0	948	A09(28952074-28953021)	97.3	A09
			A09(25510915-25511912)	98.2		A09_random(28952074-28953021)	99.1	
BjuTT5-4	ZBjuH106O08	1233	scaffold_30.1(166852-165708)	92.5	930	scaffold_30.1(64946-65862)	94.8	B
BjuTT6-1	ZBjuH058K21	1177	A07(2473977-2472803)	96.2	1179	A03(21939848-21938684)	97.7	A03
			A07(2765573-2766747)	98.0		A03(20708198-20709378)	98.9	
BjuTT6-2	ZBjuH087J23	863	A09(27193799-27193372)	96.4	1002	A09(27076762-27077609)	98.9	A09
			Un_random(93706755-93707622)	96.9		A09(23672668-23672668)	92.7	
BjuTT6-3	ZBjuH031F14	1036	A09(32538591-32537560)	97.4	963	A09(32402616-32403171)	98.6	A09
			A09_random(3439921-3440956)	98.7		A09(28629617-28630172)	99.1	
BjuTT6-4	ZBjuH022O18	1051	B03(6156413-6155347)	97.2	1068	B03(6011976-6012882)	99.1	B03
BjuTT6-5	ZBjuH143I07	1034	B08(40752199-40753230)	96.6	1115	B08(40891718-40890578)	95.6	B08
BjuTT7-1	ZBjuH159L04	950	A10(14281026-14287855)	94.3	875	A10(14428932-14428063)	99.2	A10
			A10(15377459-15378411)	95.4		Un_random(110160592-110161461)	99.1	
BjuTT7-2	ZBjuH080O14	1072	B08(28651670-28650600)	94.8	1084	B05(13990978-13992051)	97.5	B08
BjuTT3-1	ZBjuH029J10	952	A09(10801814-10802675)	99.1	902	A09(10945043-10944178)	96.0	A09
			A09_random(1204329-1205280)	99.7		A09(9183884-9184783)	97.5	
BjuTT3-2	ZBjuH183H13	973	B06(24254316-24255289)	99.9	984	B06(24372970-24371986)	99.9	B06
BjuTT18-1	ZBjuH054O02	1063	A01(6900126-6899636)	97.6	896	A01(6775183-6775797)	94.7	A01
			A04 (19187764-19188396)	97.2		A01_random(376002-376873)	96.3	
BjuTT18-2	ZBjuH181K08	1071	A03(24832511-24831559)	93.6	928	A03(24707499-24708449)	98.2	A03
			A03(23248562-23249571)	95.6		A03_random(1774070-1775012)	96.3	
BjuTT18-3	ZBjuH091K10	1151	B02(37271971-37273119)	99.5	1033	B02(36953007-36952312)	92.9	B02
BjuTT18-4	ZBjuH177N08	1179	B08(43363553-43362571)	94.8	1036	Scaffold_215.1 (85454-84890)	99.1	B08
BjuANR-1	ZBjuH082J01	981	A01(21610018-21609039)	100	890	A01(21496749-21497598)	93.7	A01
			A03_random(5751842-5752821)	98.8		A03_random(5650455-5651236)	98.1	
BjuANR-2	ZBjuH148I16	1088	A01(17599566-17600642)	98.5	996	A05(6382585-6383570)	96.7	A01
			A01_random(1627810-1628889)	97.7		C06(11884727-11885709)	94.0	
BjuANR-3	ZBjuH116E04	1136	B01(23295810-23295103)	96.8	1143	B01(22662292-22661178)	93.5	B01
BjuANR-4	ZBjuH034P21	1015	B08(31415998-31416996)	95.1	996	B08(31573791-31572839)	99.6	B08
BjuTT10-1	ZBjuH083G18	895	A02(22936601-22937747)	96.8	926	A07(287213-288132)	82.6	A02
			Un_random(20422597-20428316)	99.7		A10(7084906-7085831)	99.4	
BjuTT10-2	ZBjuH055H16	998	A06(20316953-20317946)	95.7	974	A06(20441440-20440468)	99.6	A06
			A06(20471573-20472569)	96.0		A06 (20582545-20583508)	96.9	
BjuTT10-3	ZBjuH021A16	1006	B06(30166234-30167223)	92.7	988	B06(30284319-30283311)	93.7	B06
BjuTT10-4	ZBjuH176D10	1037	B07(12155684-12156717)	99.0	1008	B07(12306990-12305968)	98.1	B07
BjuTT1-1	ZBjuH180A05	1064	A09(18800232-18799233)	96.8	888	A09(18714197-18714808)	95.7	A09
			Un_random(4883830-4884831)	96.2		Un_random(4606100-4606768)	95.5	
BjuTT1-2	ZBjuH147E23	1021	Scaffold_312.1 (121001-121983)	97.7	880	Repeat Sequence	–	B06
BjuTT2-1	ZBjuH085H24	1008	Scaffold000519(4310-5318)	99.2	933	A08(8207949-8208882)	99.3	A08
			Un_random(53133429-53134437)	99.8		A08(7146333-7147266)	99.7	
BjuTT2-2	ZBjuH034J15	1041	Scaffold_500.1 (142630-141751)	98.1	974	Scaffold_1045.1 (32982-32009)	99.2	B
BjuTT8-1	ZBjuH004L18	926	Repeat sequence	–	962	A09(15796870-15796730)	91.4	A09
			A09_random(2192731-2193692)	98.7		A09(15375879-15376928)	99.4	
BjuTT8-2	ZBjuH005J18	923	B03(8048419-8049250)	98.0	729	B03(8126536-8125803)	98.5	B03
BjuTT16-1	ZBjuH130K12	1069	A02(25075678-25075282)	84.8	1046	A02(24938143-24939195)	97.8	A02
			A02(23647099-23647956)	95.6		A02(23509181-23510233)	98.7	
BjuTT16-2	ZBjuH099A21	973	A03(20916287-20917258)	100	971	A03(21065354-21064524)	98.1	A03
			Un_random(122117253-122118230)	99.1		A03(19805901-19806859)	96.9	
BjuTT16-3	ZBjuH091L03	1089	A09(3425481-3424388)	96.9	1096	Repeat sequence	–	A09
			A09(2759676-2760769)	97.5		A04(12367561-12368716)	95.1	
BjuTT16-4	ZBjuH098G12	1116	B01(19570278-19569159)	95.7	1094	B01(19452807-19453868)	93.9	B01
BjuTT16-5	ZBjuH094N07	1014	B06(30590171-30591180)	98.3	998	B06(30721361-30720370)	95.9	B06
BjuTT16-6	ZBjuH077C18	1022	B08(41697103-41696075)	97.8	1076	B08(41543305-41544388)	97.4	B08
BjuTTG1-1	ZBjuH130K10	1077	A06(17786017-17784921)	96.1	1073	Scaffold000172(118821-119882)	96.3	A06
			A06(18565145-18566064)	94.5		Un_random(101024139-101025200)	96.8	
BjuTTG1-2	ZBjuH129A18	1083	B08(41995537-41996211)	93.2	1087	B08(42142183-42141110)	97.3	B08
BjuTTG2-1	ZBjuH088A24	1139	A01(12598037-12597696)	78.9	1145	A03(8757693-8756631)	96.4	A03
			C07(33256608-33257744)	92.1		A03(8043552-8044706)	99.1	
BjuTTG2-2	ZBjuH101A24	1047	A05(4101759-4100924)	98.8	1074	A05(3964877-3966181)	90.9	A05
			A05(3967225-3968262)	97.6		A05(3832372-3833452)	98.6	
BjuTTG2-3	ZBjuH063L13	1099	B03(32169026-32168705)	82.7	1080	B03(32059185-32060257)	96.2	B03
BjuTTG2-4	ZBjuH043G17	1094	B04(5231833-5230759)	96.8	1118	B04(5118011-5119109)	94.9	B04
BjuTT12-1	ZBjuH047J16	1005	A07(16179990-16178972)	96.9	1010	A07(16062416-16062903)	98.6	A07
			A07(14996804-14997785)	96.6		A07(14870425-14871409)	98.6	
BjuTT12-2	ZBjuH124J12	1057	B04(24922182-24923204)	90.5	1030	B04(25075022-25073994)	97.8	B04
BjuTT19-1	ZBjuH095N01	1093	Repeat sequence	–	969	A02(3047112-3048020)	96.7	A02
			Repeat sequence	–		A02(1465948-1468567)	96.5	
BjuTT19-2	ZBjuH170C22	1054	A10(11649528-11650476)	94.0	985	A10(11804011-11803026)	99.8	A10
			A10(12892359-12893412)	100		C09(43030716-43031727)	93.4	
BjuTT19-3	ZBjuH122M08	1033	B02(33362984-33362101)	92.3	888	B02(33197233-33197697)	98.5	B02

a *Position in B. rapa reference genome is listed in the first line*.

b *Position in B. napus reference genome is listed in the second line*.

### Expression of PA genes in seed coat of *B. juncea* and *B. napus*

**Figure 2:**
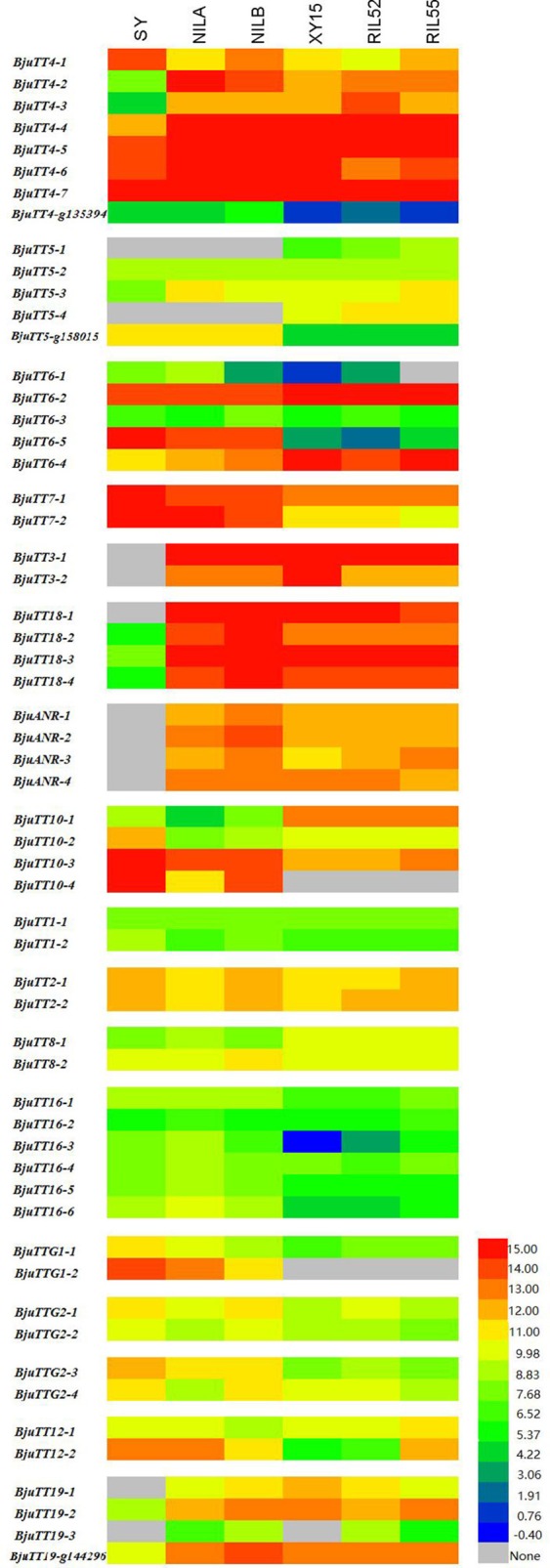
**Expession heatmap of gene expression based on FPKM data**. NILA, NILB, SY represent the seed coat of *B.juncea*, and XY15, RIL52, RIL55 represent the seed coat of *B.nupus*. The color key represents FPKM normalized log_2_ transformed counts.

**Figure 3:**
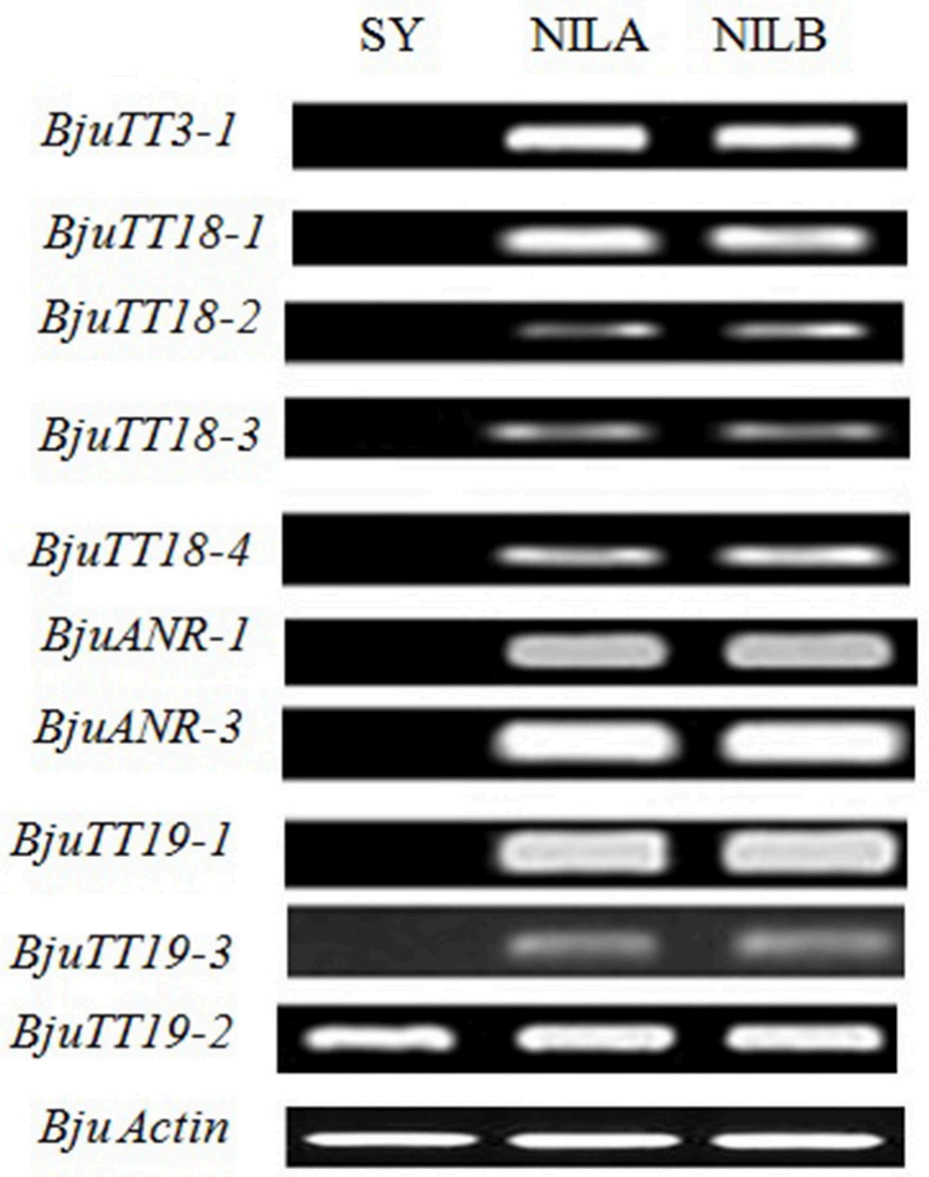
**RT-PCR analysis of genes for proanthocyanidin biosynthesis in the seed coats of ***Brassica juncea*****. SY, Sichuan Yellow; NILA and NILB, Near-Isogenic Lines A and B. Seed coats were separated from seeds at 15 days after pollination.

Fragments Per Kilobase of a transcript per Million (FPKM) analysis indicated that 55 annotated *B. napus* PA genes (excluding BnaCnng01290D and BnaA09g29340D), and all cloned *B. juncea* PA genes except *BjuTT5-1* and *BjuTT5-4* were expressed in seed coat (Figure 2, Table S7). However, transcript abundance significantly varied among PA genes, as well as accessions. In general, the expression level of structural and transporter genes were higher than that of transcriptional factor genes in black- and brown-seeded accessions analyzed. No transcripts of *BjuTT3, BjuANR, BjuTT18-1, BjuTT19-1*, and *BjuTT19-3* were detected in the seed coat of yellow-seeded SY. In addition, a 7-fold or greater difference in expression level of *BjuTT3, BjuTT18, BjuANR*, and *BjuTT19* as well as *BjuTT4-2, BjuTT4-3, BjuTT4-4*, and *BjuTT5-3* were found between SY and its brown-seeded near-isogenic lines (Figure 2, Table S7), implying that these differentially expressed genes are involved in seed pigmentation. Moreover, six additional genes, i.e., *BjuTT4-5, BjuTT6-1, BjuTT6-4, BjuTT8-1, BjuTT16-3*, and *BjuTT16-6*, were upregulated by at least 2-fold in seed coat of NILA, whereas four other genes (*BjuTT4-5, TT4_g135394, BjuTT6-4*, and *BjuTT8-2*) were upregulated by at least 2-fold in seed coat of NILB compared with SY (Figure 2, Table S7). RT-PCR analysis confirmed the differential expression profile of *BjuTT3, BjuTT18, BjuANR*, and *BjuTT19* that was carried out using FPKM analysis (Figure 3).

## Discussion

In the present study, we identified 55, 58, and 31 PA genes in *B. juncea, B. napus*, and *B. rapa* through a combination of experimental and bioinformatics approaches, analyzed their phylogenetic relationship and genomic locations in *Brassica*, and detected and compared their expression in seed coats of different accessions by RNA-seq. Cloning of these genes not only lays a foundation for the elucidation of the molecular mechanism underlying PA accumulation/profile and seed pigmentation in *Brassica* species, but also facilitates in the functional characterization of each PA gene.

The PA genes in *Arabidopsis* (16) were almost doubled in *B. rapa* (31) and nearly quadrupled in *B. juncea* (55) and *B. napus* (58). The ancestral A, B, and C genomes of the *Brassica* species contributed a comparable number of PA genes. These findings are consistent with mesopolyploid nature of *B. rapa* and the allopolyploid nature of *B. juncea* and *B. napus*, implying that polyploidization plays an important role in expansion of PA genes. However, the number of PA genes in allopolyploid *B. juncea* and *B. napus* does not amount to the sum of PA genes from both ancestral species due to gene loss by genomic fractionation during allopolyploidization. Bra036307 and Bra009770 might have been lost in *B. juncea* and *B. napus*, respectively.

Phylogenetic analysis and genomic localization of *B. juncea* PA genes indicated that 30 and 29 *B. juncea* PA genes were homologous to genes located in the A-genome chromosomes of *B. rapa* and *B. napus*, respectively (Figure S2, Table 3). However, both BESs of 23 and 15 A-genome *B. juncea* PA genes were mapped around the *B. rapa* and *B. napus* genomic position, as mapped by the full-length sequence of the corresponding genes, respectively (Table 4). The other BESs were mapped to other chromosomes or not detected in the *B. rapa* and *B. napus* reference genome. These findings indicate that although *B. rapa, B. juncea*, and *B. napus* have the common A-genome, the chromosomes of each of these species do not harbor the same structure (Zou et al., 2016). On the other hand, assembly of the present reference genomes of *Brassica* species need improving.

For 6 of the annotated PA genes in *B. juncea* GSS, no BAC clones were identified. Sequence analysis revealed that the annotated genes *ANR_g228640, ANR_g226654*, and *TT19_g167454* were false genes or artifacts that arose by misassembled sequences because these annotated genes only contain a part of the protein domains of the corresponding genes and its alignment ratios were significantly lower than other predicted genes (Table S5). No BACs carrying the annotated gene *TT4_g135394, TT5_g158015*, or *TT19_g144296* were detected, most probably because the sequenced fragments amplified from positive BACs were too short to distinguish different members of a gene family (Table 1), or maybe because the primers used in screening the BAC library were not appropriate. In contrast, the cloned *BjuTT4-1, BjuTT4-7*, and *BjuTT16-5* genes were not predicted from our GSS dataset, illustrating that these genes were missed in our genome sequence survey of *B. juncea* genome, most probably because of insufficient sequencing depth or assembly errors.

In *Arabidopsis*, three additional PA genes *TT15* (DeBolt et al., 2009), *TT9* (Ichino et al., 2014), and *TT13/aha10* (Appelhagen et al., 2015) have recently been cloned. Their *Brassica* homologs were not investigated in the present study. In our next study, we will clone and analyze these genes to complete the set of PA genes in *Brassica* spp. Initial screening of our BAC library identified seven BAC clones for each of these three genes. Sequencing of the fragments amplified from these BACs is underway.

RNA-seq and FPKM analyses showed that BnaCnng01290D, BnaA09g29340D, *BjuTT5-1* and *BjuTT5-4* were not expressed in the seed coat, indicating that these genes might not be involved in seed pigmentation. Interestingly, the *BjuTT3, BjuTT18*, and *BjuANR* genes were not expressed in yellow-seeded testa, but expressed very high in brown-seeded testa of *B. juncea* (Figure 2, Table S7), which is consistent with previous results (Yan et al., 2008, 2011; Akhov et al., 2009; Liu et al., 2009, 2013; Jiang et al., 2013), suggesting that seed color is determined by expression of genes that encode enzymes that catalyze PA biosynthesis. Concomitant with the absence of expression of these enzyme-encoding genes in yellow-seeded testa, the early stage genes, *BjuTT4-2* and *BjuTT4-3*, which encode chalcone synthase, and transporter genes, *BjuTT19-1* and *BjuTT19-3*, which encode glutathione transferase, were remarkably downregulated or not expressed in yellow-seeded testa (Table S7). These findings illustrate that these genes are co-regulated with *BjuTT3, BjuTT18*, and *BjuANR*, and their expression is not essential to the production of biosynthetic substrates and epicatechin transport in yellow-seeded testa. Other *BjuTT19* and *BjuTT4* genes did not show differential expression between yellow- and brown-seeded testa (Figure 2, Table S7), implying that these genes are not involved in seed pigmentation and that their biological roles require further investigation.

To answer the questions why all the *BjuTT3, BjuTT18*, and *BjuANR* genes are not fully expressed in yellow-seeded testa and why these genes are mutated, transcriptionally regulated, or both, we also cloned full-length genomic sequences of these genes from SY and compared them with the corresponding sequences from PM. Comparative analysis showed no differences, except for a 33-bp and 2-bp difference in *BjuTT18-2* and *BjuTT3-1*. In *Arabidopsis*, the genes *TT3, TT18*, and *ANR* are transcriptionally regulated by TT2-TT8-TTG1 complex (Xu et al., 2013). Comparison between SY and PM uncovered a 1275-bp insertion in exon 7 of *BjuTT8-1* and a C-T transition in exon 7 of *BjuTT8-2* of SY, which is almost in agreement with findings from Padmaja et al. (2014) who speculated that the *TT8* gene controls seed pigmentation in *B. juncea*.

## Conclusions

A total of 55 genes homologous to 16 *Arabidopsis* proanthocyandin-associated genes were identified and cloned from *B. juncea*. Approximately 58 and 31 PA genes were detected in *B. napus* and *B. rapa* genome databases. Around 30 of these cloned *B. juncea* genes were located in the A-genome chromosomes, except A04, whereas the remaining 25 were mapped to the B-genome chromosomes, except B05 and B07. A majority of these genes were expressed in the seed coat of *B.juncea* and *B. napus*. Tissue-specific expression of the *TT4, TT5*, and *TT19* genes were observed in *B. juncea* and *B. napus*. *BjuTT3, BjuTT18, BjuANR, BjuTT4-2, BjuTT4-3, BjuTT19-1*, and *BjuTT19-3* were transcriptionally regulated in the seed coat and not expressed or downregulated in yellow-seeded testa. In summary, the present study facilitates in better understanding the molecular mechanism underlying PA accumulation/profile and seed pigmentation, as well as in further characterization of the structure, variations, and functions of PA genes in *Brassica* spp.

## Author contributions

ZL and CG designed the research. XL, YL, MY, and DS performed the research and analyzed the data. XH took part in screening of the BAC library. SL and SC provided the genes primers and assisted with sequencing of the BAC clones. ZL and XL wrote the manuscript. All authors read and approved the final manuscript.

## Funding

This work was supported by the National Natural Science Foundation of China (No.31101176 and No.31271762).

### Conflict of interest statement

The authors declare that the research was conducted in the absence of any commercial or financial relationships that could be construed as a potential conflict of interest.

## Supplementary material

Figure S1 **The PCR screening systems and process of BAC library**. Screening of a specific clone by five-step PCR is shown: Step 1, screening of 19 superplates: A positive signal was detected in the superplate3 (plates SP 021–030). Steps 2–4, screening against superplate3 by 3D-PCR: Positive signals were identified in 1D, 2D, and 3D; these consisted of Plate034, C15&16, and RI&J, respectively, indicating that Plate034, column 15/16, and row I/J contained the specific BAC DNA. Step 5, screening of four candidate BACs: A positive signal was detected in the one of the four candidate BACs (ZBjuH034I15, ZBjuH034I16, ZBjuH034J15, and ZBjuH034J16). Consequently, a BAC clone containing the specific sequence was identified as the clone of ZBjuH034J15.

Figure S2 **Phylogenetic trees of proanthocyanidin-associated genes from ***Brassica juncea, B. rapa, B. napus***, and ***Arabidopsis thaliana*****. Phylogenetic reconstruction of proanthocyanidin biosynthetic genes from *Brassica juncea, Arabidopsis thaliana, B. rapa*, and *B. napus*. Phylogenetic trees were constructed from genomic sequences of *PA* genes using Neighbor-Joining (NJ) algorithm and 1000 bootstrap replications provided in MEGA5.2. **(a)** TT4; **(b)** TT5; **(c)** TT6; **(d)** TT7; **(e)** TT3; **(f)** TT18; **(g)** ANR; **(h)** TT10; **(i)** TT1; **(j)** TT2; **(k)** TT8; **(l)** TT16; **(m)** TTG1; **(n)** TTG2; **(o)** TT12; **(p)** TT19.

Figure S3 **Sequences of annotated but unidentified proanthocyanidins-associated genes in ***Brassica juncea*****.

Table S1 **Sequences of the primer pairs used in screening for proanthocyanidin-associated genes of ***Brassica juncea*** BAC library**.

Table S2 **Sequences of the primer pairs used in expression analysis of proanthocyanidin-associated genes of ***Brassica juncea*****.

Table S3 **Global statistics of the genomic assembly of ***Brassica juncea*****.

Table S4 **Annotated genes of ***Brassica juncea*** genome survey sequences (xls)**.

Table S5 **Annotated proanthocyanidin-associated genes from ***Brassica juncea*** genome survey sequences (xls)**.

Table S6 **Mapping to the GSS sequence of full-length sequences of ***Brassica juncea PA*** genes cloned in this study (xls)**.

Table S7 **Transcript abundance of proanthocyanidin-associated genes in the transcriptome of ***Brassica napus*** and ***B. juncea*** seed coats**.
