# Supplementary figures and images for: Genome-Wide Identification, Localization, and Expression Analysis of Proanthocyanidin-Associated Genes in Brassica
Source: Front Plant Sci. 2016 Dec 9;7:1831. doi: 10.3389/fpls.2016.01831 (PMC5145881; doi:10.3389/fpls.2016.01831)

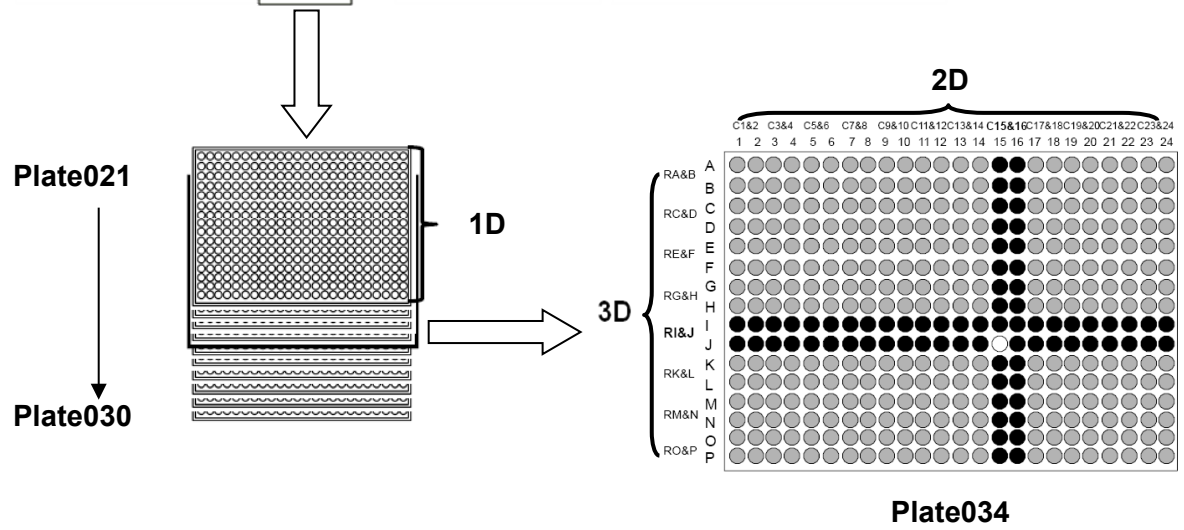

Supplement: Figure S1 — The PCR screening systems and process of BAC library. Screening of a specific clone by five-step PCR is shown: Step 1, screening of 19 superplates: A positive signal was detected in the superplate3 (plates SP 021–030). Steps 2–4, screening against superplate3 by 3D-PCR: Positive signals were identified in 1D, 2D, and 3D; these consisted of Plate034, C15&16, and RI&J, respectively, indicating that Plate034, column 15/16, and row I/J contained the specific BAC DNA. Step 5, screening of four candidate BACs: A positive signal was detected in the one of the four candidate BACs (ZBjuH034I15, ZBjuH034I16, ZBjuH034J15, and ZBjuH034J16). Consequently, a BAC clone containing the specific sequence was identified as the clone of ZBjuH034J15. [file Image1.PDF]

a

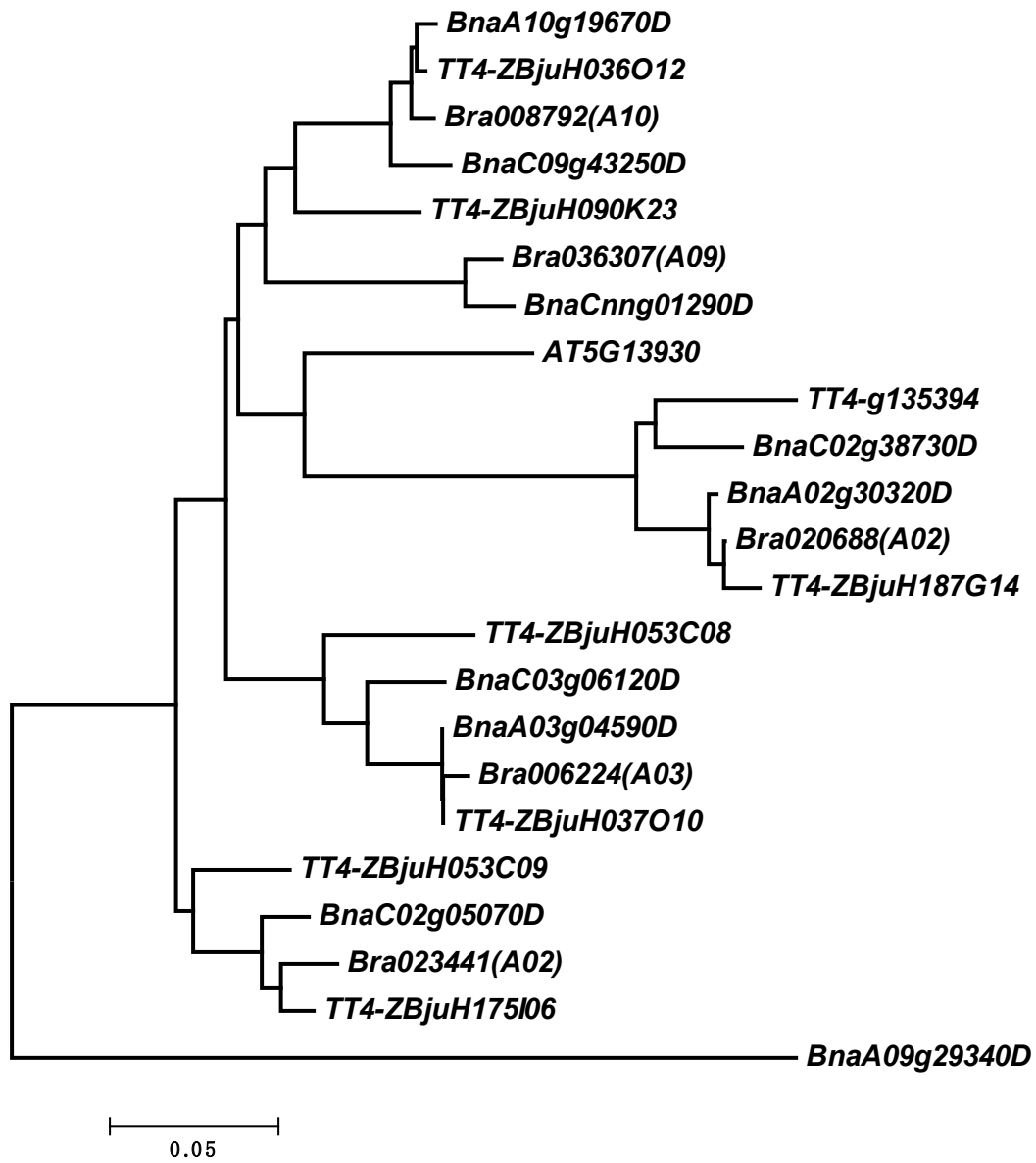

b

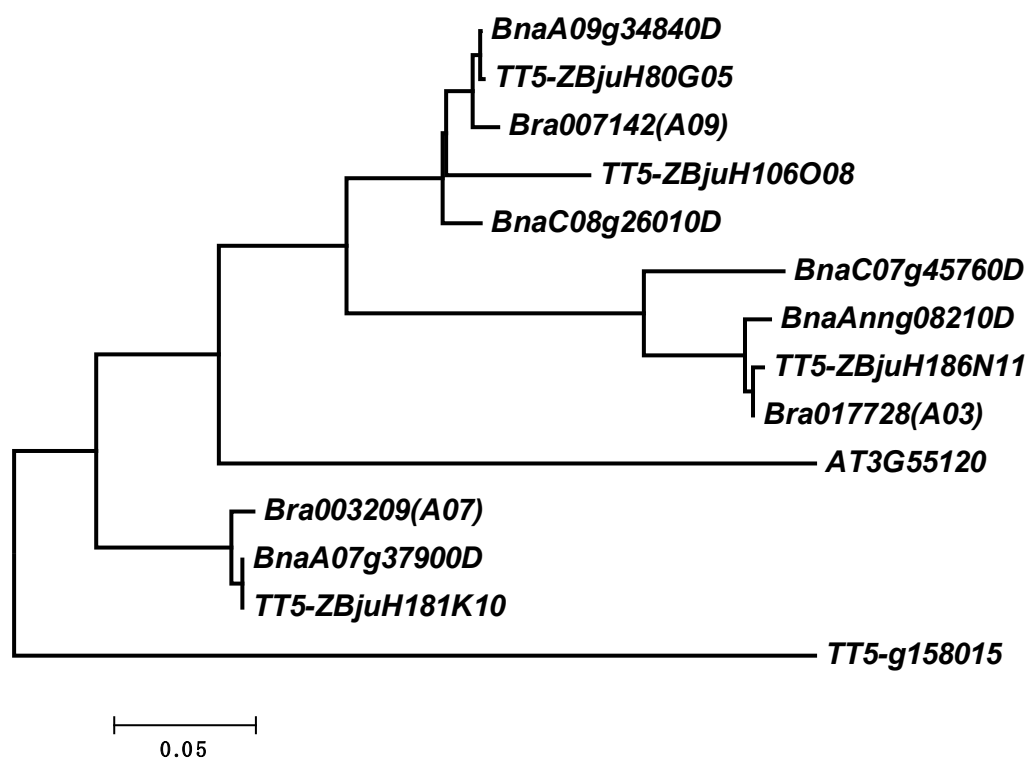

c

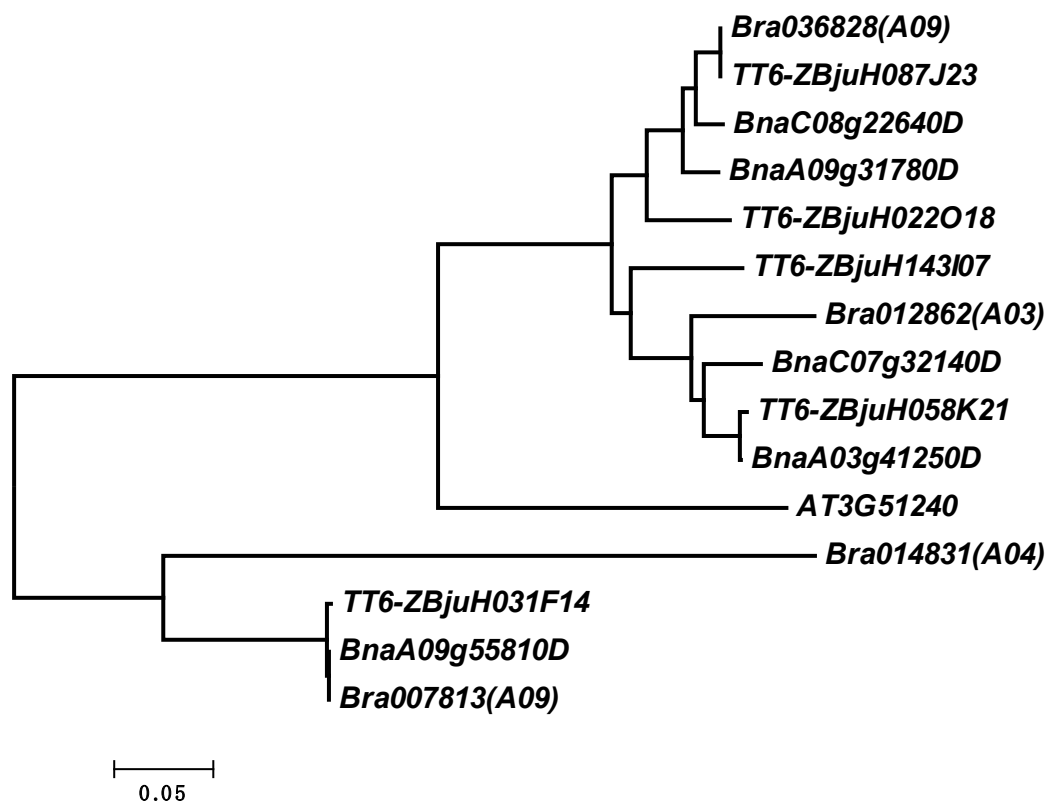

d

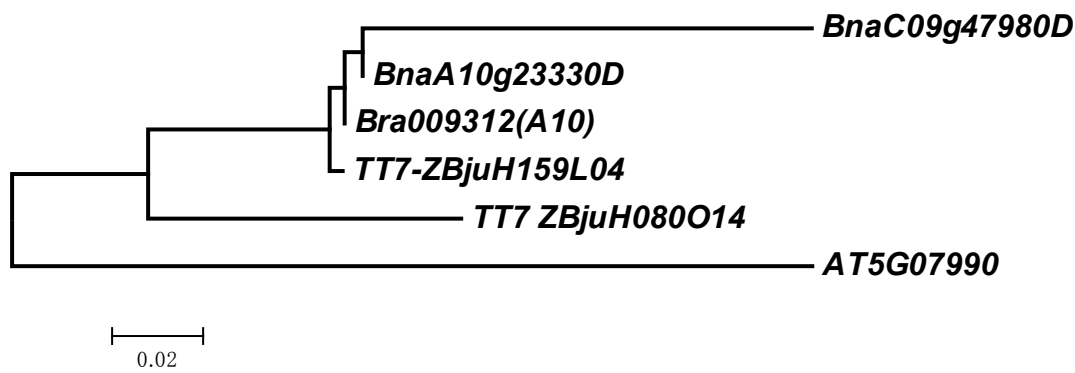

e

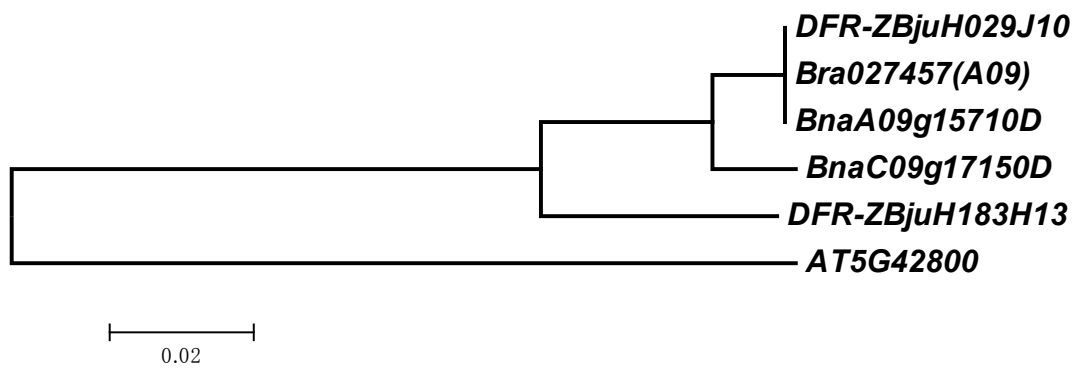

f

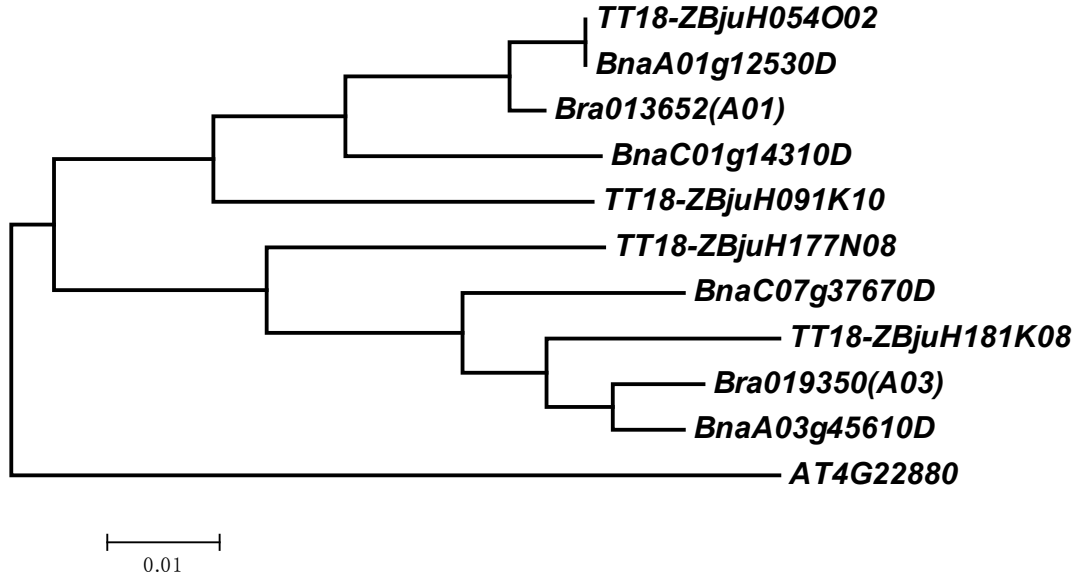

g

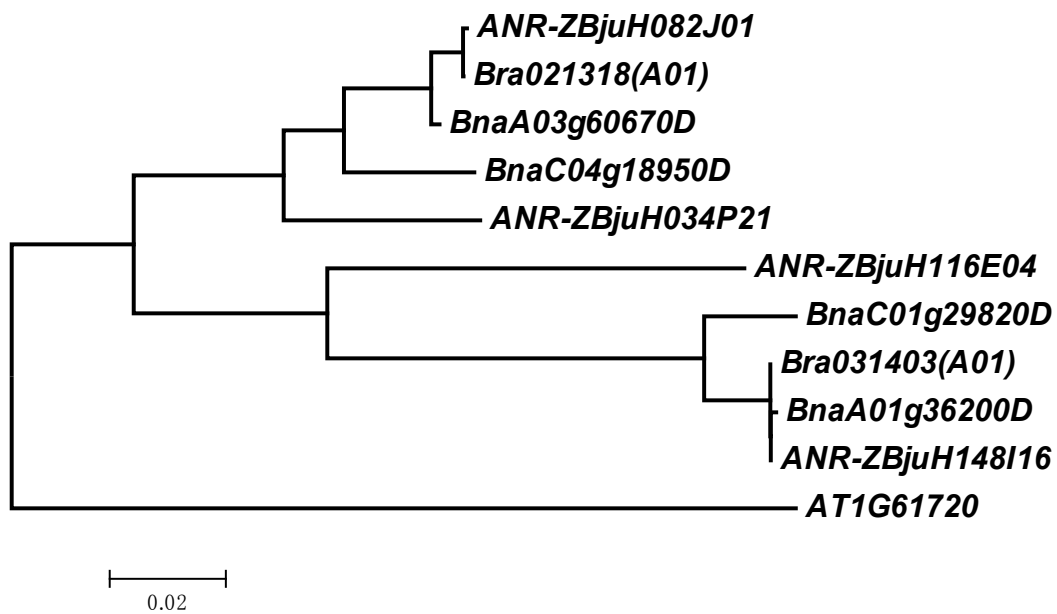

h

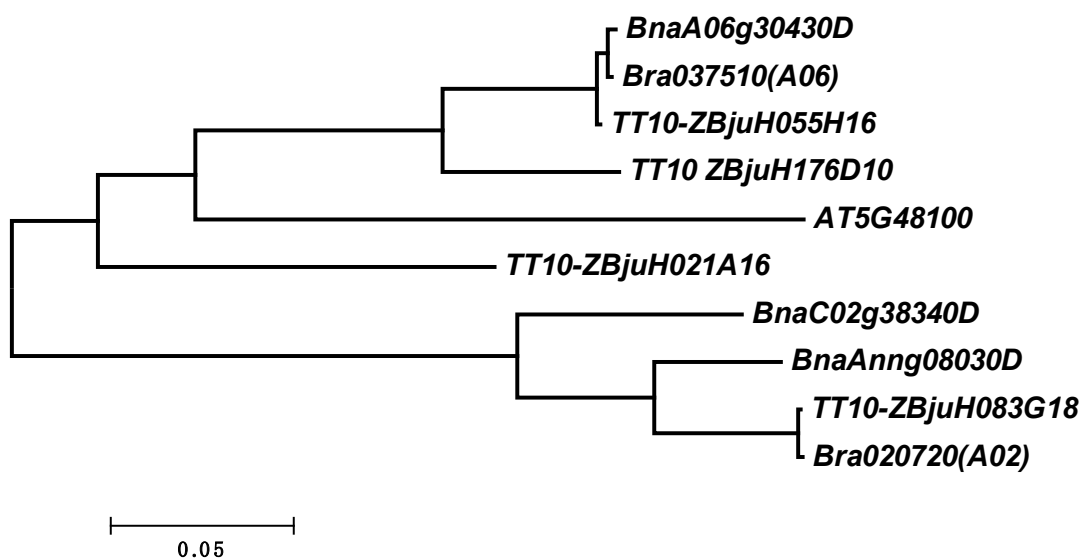

i

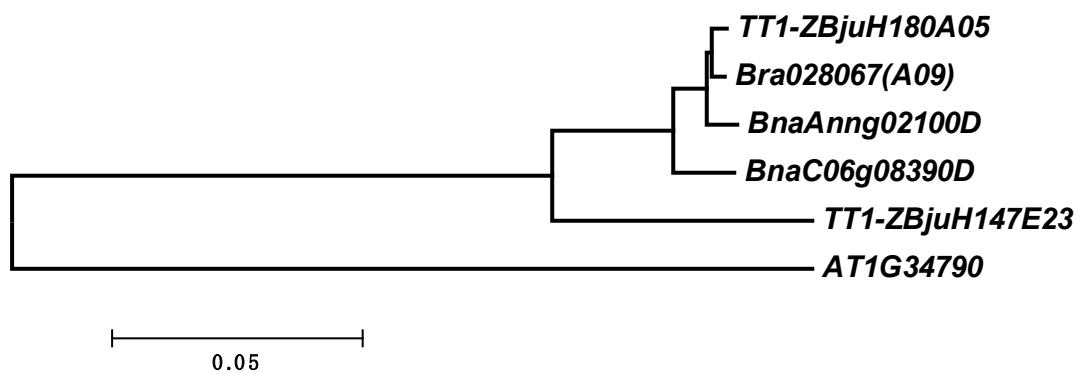

j

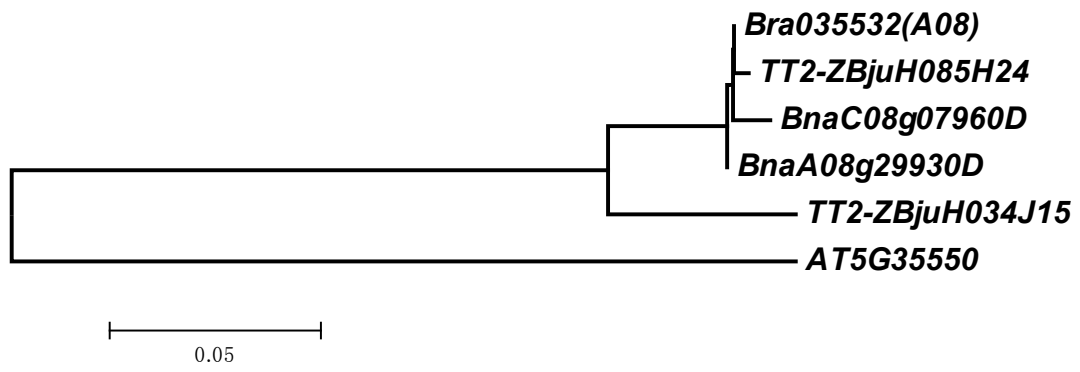

k

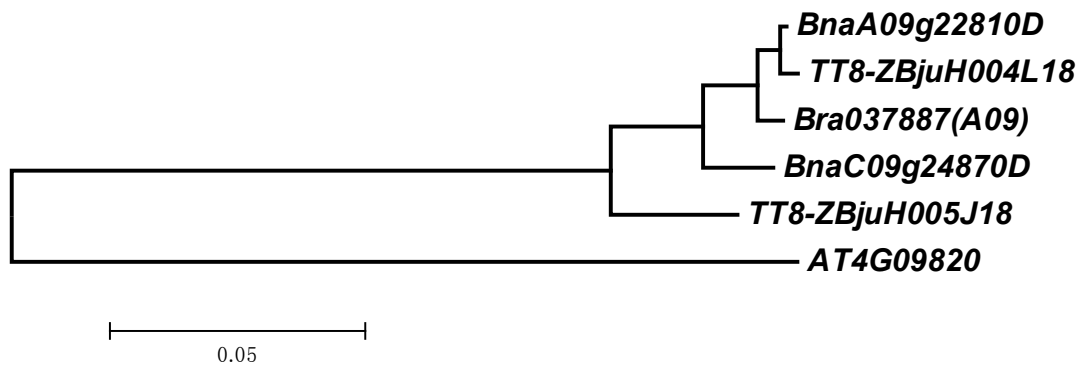

I

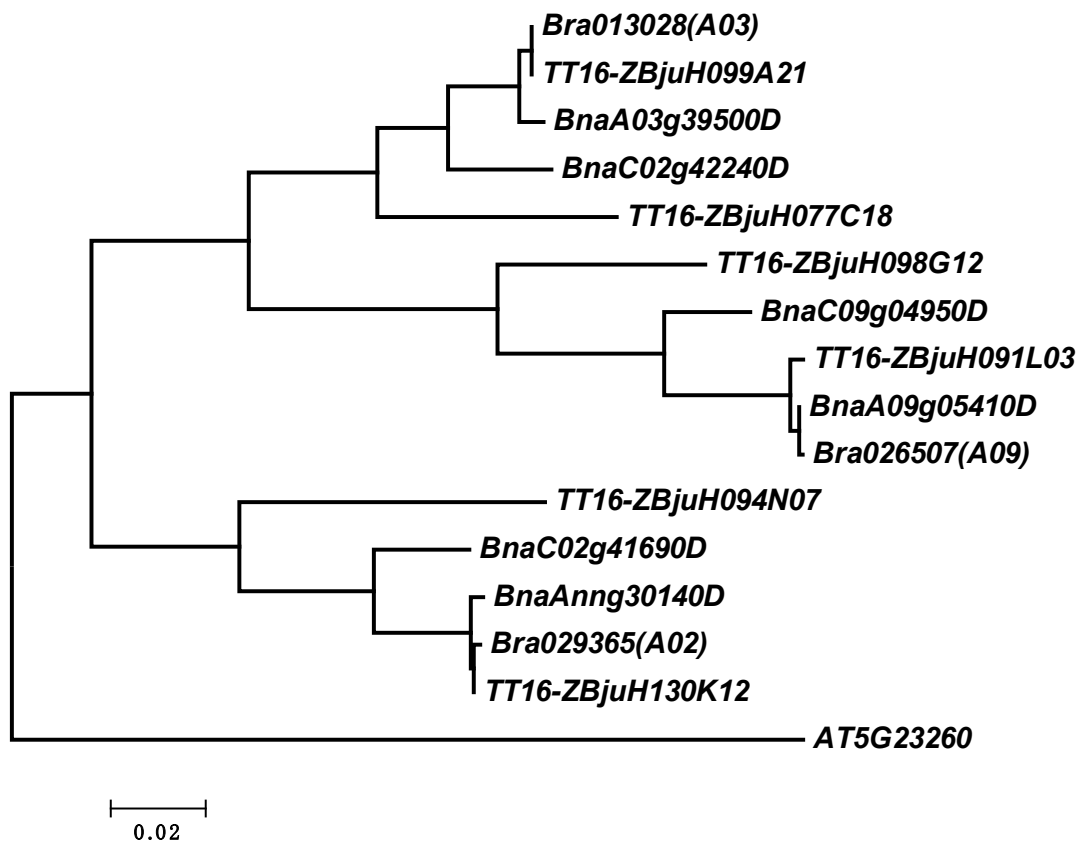

m

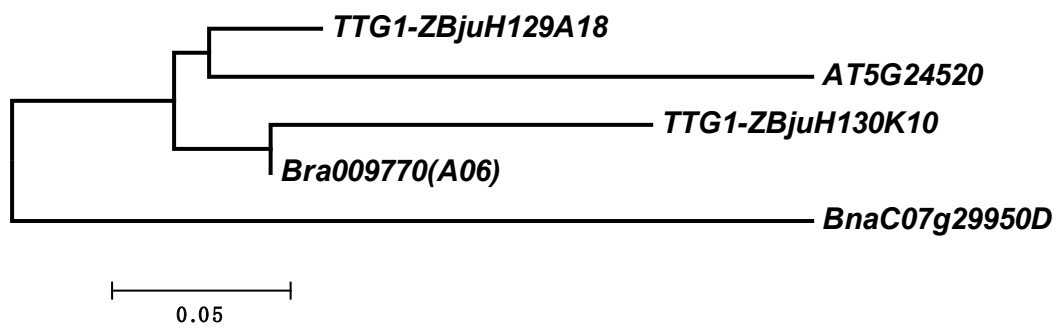

n

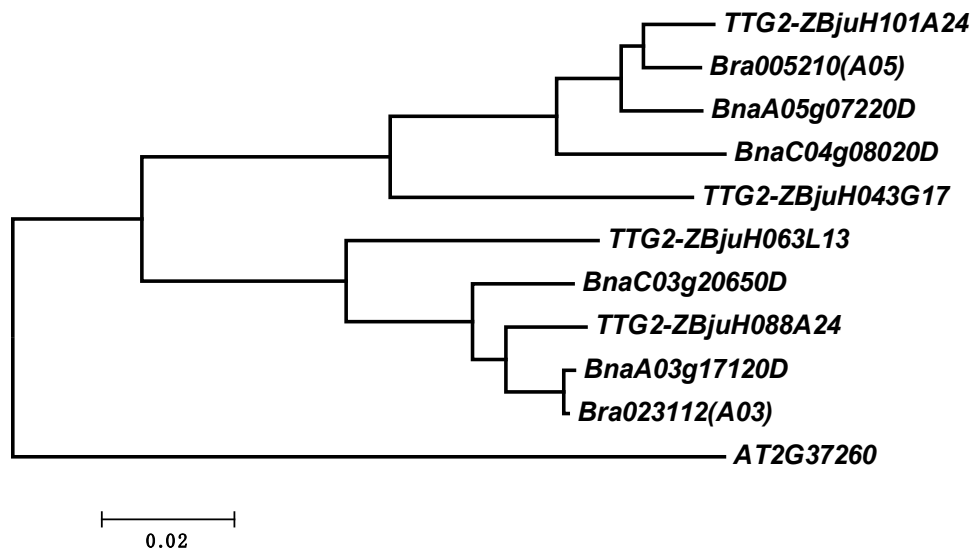

o

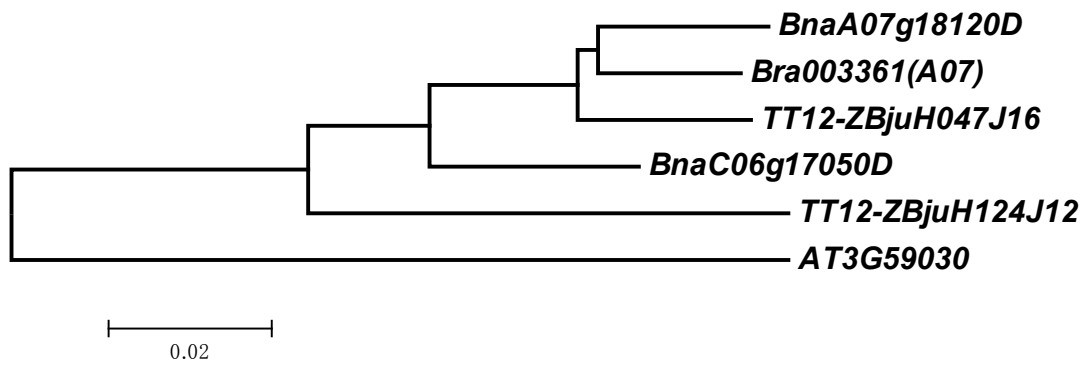

p

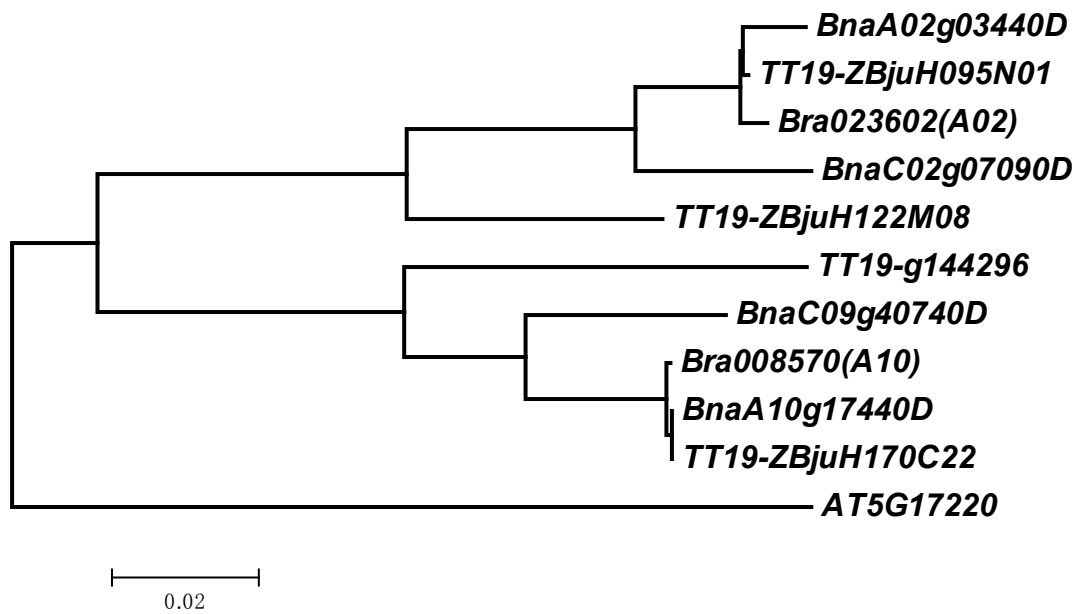

Supplement: Figure S2 — Phylogenetic trees of proanthocyanidin-associated genes from Brassica juncea, B. rapa, B. napus, and Arabidopsis thaliana. Phylogenetic reconstruction of proanthocyanidin biosynthetic genes from Brassica juncea, Arabidopsis thaliana, B. rapa, and B. napus. Phylogenetic trees were constructed from genomic sequences of PA genes using Neighbor-Joining (NJ) algorithm and 1000 bootstrap replications provided in MEGA5.2. (a) TT4; (b) TT5; (c) TT6; (d) TT7; (e) TT3; (f) TT18; (g) ANR; (h) TT10; (i) TT1; (j) TT2; (k) TT8; (l) TT16; (m) TTG1; (n) TTG2; (o) TT12; (p) TT19. [file Image2.PDF]
