# Supplementary material for: Genome-Wide Identification, Localization, and Expression Analysis of Proanthocyanidin-Associated Genes in Brassica
Source: Front Plant Sci. 2016 Dec 9;7:1831. doi: 10.3389/fpls.2016.01831 (PMC5145881; doi:10.3389/fpls.2016.01831)
Supplement: Figure S3 — Sequences of annotated but unidentified proanthocyanidins-associated genes in Brassica juncea. [file Image3.PDF]

>TT4-g135394

1 ATGGTATTGA CGGGTGCCTT ATCGTTGGAT GAGATCAGAA AGGCACAAAG AGCTGATGGA

61 CCAGCAAGTA TCTTGGCCAT AGGCACTGCC AATCCTCCAA ACCAAGTGAT CCAAGCAGAG

121 TATCCAGACT ACTACTTCCG CGTCACCAAC AGTGACCACA TGACCGACCT CAAGGAGAAG

181 TTCAAGCGCA TGTGTAAGTC TTCATTTTCA TTTTGCCACA CCTATACTCA TTTATATGTA

241 TACCTGAACT TTTTCTATTA CTATAACAGG CGACAAATCA ATGATAAGGA AACGTTACAT

301 GCACTTGACC GAGGAGTTTC TCAAGGACAA CCCTAACGTA TGCGCCTTCA TGGCTCCTTC

361 CTTGGACGTT AGGCAGGACA TCATGGTGAC CGAGGTCCCT AAGCTAGGGA AAGAGGCCGC

421 GGTGAAGGCT ATCAAGGAGT GGGGGCAGCA CAAGTCCAAG ATCACTCATC TAGTCTTCTG

481 CACCACCTCT GGTGTCGACA TGCCTGGTGC TGACTACCAG CTCATCAAGC TCCTTGGCCT

541 CTCTCCTTCT GTTAAGCGTG TCATGATGTA CCATCAAGGT TGCCACGCCG GTGGGACTGT

601 TCTCCGTTTG GCTAAGGACA TAGCTGAGAA CAACCGCGGC GCTAGAGTAC TTATTGTTTG

661 CTCTGAGATT ACTGTGGTTA CCTTCCGTGG GCCCTCTGAG ACCCACCTTG ACTCCCTCGT

721 TGGGCAGTCT CTTTTTGGTG ACGGTGCGGC TGCACTCATT GTGGGTTCAG ATCCTGACAC

781 CACTGTCGGA GAGAAACCAA TCTTTGAGAT GGTCTCTGCT GCACAGACCA TCCTTCCTGA

841 CTCTAAAGGA GCCATAGAAG GACATCTGAA GGAGGTGGGA CTCACTTTCC ATCTCTTGAA

901 GACCGTCCCC GGTCTCATCT CAAAGAACAT TCAAAAGAGT CTTGAAGAAG CGTTTAAACC

961 GCTGGGGATA AGCGACTGGA ACTCCCTCTT CTGGATAGCT CACCCTGGAG GCCCTGCCAT

1021 CTTGGACCAC GTTGAGATAA AGCTAGGACT AAAGGCAGAG AAGATGAAGG CCACCCGTCA

1081 TGTCCTGAGC GAGTATGGAA ACATGTCCAG TGCCTGTGTC TTCTTCATAC TTGACGAGAT

1141 GAGGAGGAAG GCGGTAGAGG ATGGTGCAAA GACGACAGGA GAAGGGTTGG AGTGGGGTGT

1201 CTTGTTTGGT TTTGGACCAG GTCTCACTGT AGAGACAGTA GTCTTGCACA GCGTTCCGCT

1261 CTAA

>TT5-g158015

1 ATGTCTTCTT CCCACTGTCC GTCACCGTTA CCCTCCGTCA CCAAACTTCA AGTAGACTGC

61 GTTACTTTTC CACCGTCCGT CATCTCACCG GCCTCCTCCA ATCCCCTCTT CCTCGGTGGC

121 GCAGGTTCGA ATACGCTTGC TTTCTTTTTT TTTTTTTCGT TTTAATCTAT GATGTTTATT

181 TGTTATATTT CTACGCAGGT GTGCGAGGTT TGGATATCCA CGGAAAATTC GTGATCTTCA

241 C**CGTCATTGG AGTCTACCTA G**ATGCTGTCT CCGTCCCGTC ACTCTCCGTT AAGTGGAAAG

301 GCAAAACCAC AGAGGAGCTA ACGGAGTCCG TCCCTTTTTT CCGTGAAATC GTTACAGGTG

361 CTTTACTTAC ACCACTTACT TAAGTACCAC GTCATGTCAA TGTGTTTCAA TTAAAAGGGG

421 TTCTACTTTG TTTTGACCAA TGTGCTAAGC GGGGGTGAGT GACTCCATCC TTTAACACCT

481 AGAAGGCTAG AACGATTAGT TGGGAACTTA GATTGTTAGT CTAATCATTT ATAAATTATT

541 ATAATATTCT AAGTATTTAT CATGATTTAT AAATAATATT AATATATTAT TTTGTTTTGC

601 TAAATTTTAG ATTATAATAT TATTAATAAT AATAATTGAT AAAATAAAAG TTATATTACT

661 TATTATTATA ATATTATATT TTCTATGTGG TTTAGAAGGC AATAGCATGT TTTAGAGGGT

721 CTGGTCTTGA CCGCTATTAA TTAATTACCT TTTTGTTTAA TTCTTAATTA GGTTCGTTTG

781 AGAAGTTTAT CAAGGTTACG ATGAAACTGC CGTTAACGGG ACAGCAATAT TCGGAGAAAG

841 TAACAGAGAA CTGTGTGGCG ATTTGGAAAT CGTTAGGTAT CTACACAGAC GCTGAAGCTA

901 AAGCTGTGGA GAGGTTTTTG GAGGTCTTCA AGGACCAAAC TTTCCCTCCT GG**TGCTTCGA**

961 **TCCTCTTTGC TC**TCTCCCCT AACGGCTCTC TCATGGTATA TACTCACAAA ACTTCTTTAT

1021 AACATTGACT TGCGTTTGCG TTTACATGTT ACATTAGTAT GATTTTTTTC TTACATATAA

1081 AACCATAGTT TTTGTCATCG TAAATGCAAT CGTGACCAAC TGTTATTATC TCAACTGGAA

1141 AAATACTATA CGCAAANNNN NNNNNNNNNN NNNNNNNNNN NNNNNNNNNN NNNNNNNNNN

1201 NNNNNNNTCA CTTTTTTTAA AAAAAAAAAA AGAAGAAGAT TGTTTACATA GCTAATGTAG

1261 AGATGACGTT TGAAACATGT ACATTTGAGT AAAAATTGTG TTTTCAATTT TCGTTAAGTT

1321 GTTTAGGTGT GGACGAAAGA AACTATTGTA GTACATACGT GATAGCTTTG AAAAAACTTT

1381 ATGACTCTGT TTGTTGTGAA TTAAAATGTT TGCAGATTGC GTTTTCCAAA GACGATAGTA

1441 TTCCTAAAAC CGGAAAAGCC GTGATCGAGA ATAAATTGTT GGCAGAGGCA GTCCTTGAAT

1501 CGATCATAGG AAAGAAGGGT GTGTCTCCTG GGGCTAGGTT GAGTCTTGCC GAGAGATTAT

1561 CTCACCTGAT GAAGGAGAAC AAGGTCAAAG AAGATGCAAC AAAGACTGAT AATCAAGATG

1621 AAGCTAACGA TCTCTCCCTT GGAGATAAGT TGGCCAAAGA GAACTGA

>TT19-g144296

1 ATGGTTGTGA AAGTATACGG ACAGGTTACT GCAGCTTGTC CGCAAAGAGT CTTGCTTTGT

61 CTTCTTGAGA AAGAAATTGA TTTTGAGATT GTTCACATCG ATCTTGATAC ATTGGAGCAG

121 AAAAGACCAG AACATCTTCT TCGTCAGGTA AACGTTTCAT GTTTTTTTTT CTTCTAAAGT

181 ATAAACAAAC CATTAGTATA ATTAACTGAT GTTTTCTTGT AGCCATTTGG TCAAGTTCCA

241 GCCAT**AGAAG ATGGAGACTT CAAGCTTT**TT GGTTAAGGAT CCTTACACCA AAAATTTTAA

301 AACACTTCTT GTAAGACTTG GTTTCTAATC ATTTATATTA TGTGTTTTGA TATTTCGCGT

361 GTTTTGCCCA AAGAATCTCG AGCCATTGCG AGATACTACG CGACCAAGTA CGCGGACCAA

421 GGCACGGACC TTTTGGGAAA GTCTCTAGAG CACCGAGCCG TCGTGGACCA GTGGGCTGAT

481 GTTGAGGTGG GTTACTTCAA CGTTCTGGTC CACCCTATTG TGATGAACCT AGTCATCAAG

541 CCCAGGCTAG GCGAAGAATG TGACCTCACT GAGGTGATAG AGCTCAAGGT GAAGCTGGAG

601 GTGGTCCTGG ACATATACGA CAACCAGCTT GCTTCGAACC GGTTTTTGGC TGGTGATGAA

661 TTCACTATGG CTGATTTGAC GCACATGCCG GCGATGAGGT ACT**TGATGGG TACCGGTCTA**

721 **AAC**CGGATTG TTAAGGATCG GGTGAATATG AACCGGTGGT GGGAAGAGGT TACGGCTAGA

781 CCGGCCTGGA AGAAGCTTAT GAAAATGGCT GGTTTTTCGA ATTAG

ANR_g226654

1 AGGATCTTCA AGGCAGACTT GACCGATGAA GGGAGTTTCA GTTTACCAGT CTCGGGAAGT

61 GAGTATGTTT TCCATGTCGC AACGCCAATC AGCTTTGCAT CTCAAGATCC CGAGGTCTGT

121 TTGTGCATAC TTTTTAACTC TAAATAAAAA CGTTTTCAGG CTACATATCT TGTTTGTGGT

181 TCAATGGTAC AGAAAGACAT GATCAATCCT GCGATACAAG GAGAGATCAA CGTGTTGAAA

241 TCTTGCTTAA ACTCGAACTC AGCCAAGCGC GTGATCTACA CTTCTTCAGC TGCTGCGGTT

301 TCTATCAACA ATCTTTCTGG ACCTGGACTT GTGATGACCG AAGAAAACTG GTCTGATATT

361 GATTTTCGCA GAAAGGAGAA ACCGTTTAAC TGGGTAATAA CAACTACTTG CTACACAAGT

421 TAGGTTTCTT TTACCAAGTT CACGTACTTC ATGTCTCTGT TATTTTTTAG GGTTACCCTA

481 TCTCAAAGGT GTTAGCAGAA AAGGCAGCTT ATACATTTGC GAAAGAGAAC AAAATCGATC

541 TCGTTACCCT GGTTCCAGCA CTCATAGCTG GAAACTCTCT CCTCTCTGAT CCTCCGAGCA

601 GTTTATCTCT CTCGATGTCT ATCACTAGTA AACATGAACT TTCTATTTGA CCCTTTCTAT

661 TAACGTTTCA CGCATGGGCG GATCTAAAAA TATTTTCCGT CGGAGGCACA AAAATAAAAA

721 CAAC

>ANR_g228640

1 ATGACAACAA TCACTGATCA GACCGCTGTA ACCACCGGAA CAAAGAAGGT TTGTGTCATC

61 GGTGGCACAG GAAACTTAGC CTCTATTCTC ATCAAGCATC TGCTTCTTAA TGGCTACAAA

121 GTTAACACCA CCGTTAGAGA TCTAGGTTCT TCATTTCTTT CTTGTCTCAA GTCATTTTGA

181 ATGTTAACTG ACTCTTTTTA TTCTAAAATT GCAGATAATG AGAAGAAAAC TGCTCACCTT

241 AGGATACTTC AAGATCTCGG GGAACTTAGG GTCTTCGAGG CGGACTTGAC CCATGAAGGG

301 AGTTTCAATT CACCAGTCTC GGACTGTGAA TATGTTTTCC ATGTCGCAAC ACCAATCAAC

361 TTTACATCTC AAGATCCCGA GGTCTGGTCT TTAACATATT TGTCCCTACT TTTTAACTCT

421 AAGAAAAACA TATCTTGTTT GGTGTTTCAT ATGTACAGAA AGACATGATC AATCCAGCGG

481 TACAAGGAGT GCTCAACGTG TTGAGATCTT GCTTCCTCTC GAACTCAGTC AAGCGCGTGA

541 TCTACACTTC TTCAGCTGCT GCGGTTTCTA TCAACAATCT TTCAGGACCT GGACTTGTGA

601 TG

>TT19-g167454

1 ATGGTTGTGA AACTATACGG ACAGGTAACT GCAGTTTGTC CACAAAGAGT CTTGCTTTGT

61 TTTCTGGAGA AAGAAATTGA GTTTGAGATT GTTCATGTCG ACCTCGATAC ACTAGAGCAG

121 AAGAAACCAG AACATCTTCT TCGTCAGGTA GACTGTTTAG TATCTTCCTA ACTATTGAAA

181 CAAACCATGT GATGAATATA TTTAGTATAA TTATTGTTTA TTTTTTGGTA GCCATTTGGT

241 CAAGTCCCAG CCATAGAAGA TGGAGATTTA AGCTTTTTGG TAACGATTCT AAGTTTAAAA

301 ACTTTTTTTT TCAAGACTTG TTCTCTAATC ATTTATGCAT TGTGTTTTGA AGAATCTAGA

361 GCCATTGCGA GATATTACGC GACCAAGTAC GCGGACCAAG GCACGAACCT TTTGGGCAAG

421 TCTCTAGAGC ACCGCCGAGC CATCGTAGAC CAGTGGACCG ATGTGGAGAC CCATTACTTT

481 AACGTTCTGG TCTTCCCCAT TGTGCTTAAC CTAGTCATTA AGCCCAGGTT AGGCGAAGAA

541 TGTGACGTCG TTTTGGTCGA GGAGCTGAAG GTCNGGTGGT CTTGGACATA TACGAGAACC

601 GGCTTGCTTC GAACCGGTTT TTGGCTGGTG ATGAATTCAC TATGGCTGAT TTGACGCACA

661 TGCCGGCGAT GGGGCAACAT CAAAGTATGT GAGGAACCTT GACCGGATGT GTCGATCAAG

721 GGGAAGTGTT AAAGATAACT TGGTGATCGA TCCATGAACA GGTCCCTACC GCGCAGACCC

781 ATCTGGCCCA GTCCATAG
