# Supplementary material for: Genome-Wide Identification, Localization, and Expression Analysis of Proanthocyanidin-Associated Genes in Brassica
Source: Front Plant Sci. 2016 Dec 9;7:1831. doi: 10.3389/fpls.2016.01831 (PMC5145881; doi:10.3389/fpls.2016.01831)
Supplement: Table S1 — Sequences of the primer pairs used in screening for proanthocyanidin-associated genes of Brassica juncea BAC library. [file Table1.DOC]

Table S1 Sequences of primer pairs used in screening for proanthocyanidin-associated genes of *Brassica juncea* BAC library.

| Gene | Primer name | Forward sequence (5’ → 3’) | Reverse sequence (5’→ 3’) |
| --- | --- | --- | --- |
| *BjuTT4* | STT4 | AAGGAGAAGTTCAAGCGCATG | CTCTTTGCCTAGCTTAGGGAC |
| *BjuTT5* | STT5 | CGTCATCGGAGTTTACCTTG | GAGCGAAGAGGATGGAAGCA |
| *BjuTT6* | STT6 | GAAGGGTGGGTGAAAGTGAC | CAAAGTGATGGTTCCAGGGT |
| *BjuTT7* | STT7 | ATCAACGGCTATCATATCCC | CGTAAGCACTCCTATCCAAC |
| *BjuTT3* | STT3 | CCATCATAAGACAAGGACAG | GTAAAGAGACAGGGAGAAAA |
| *BjuTT18* | STT18 | TTCTACGAGGGTAAATGGATC | CACTCACCATCTCAGGCAACG |
| *BjuANR* | SANR | CAACAACCTTTCGGAACCTG | AACAGCAAATGTAGCGACCA |
| *BjuTT10* | STT10-1 | ACCCTAACATCAACTTCAACA | CACCACCAACTAAACTCGTC |
|  | STT10-2 | GTGTGAAGGTCCAAACGGGT | GGAGTCTGCAAAAACAACGG |
| *BjuTT1* | STT1 | CGAGGTAGAAGATGAAGAGG | TATCAAGATCCAGAGGGAAT |
| *BjuTT2* | STT2 | GCGAACAGACAATGAAATAA | GGTCTAACAATTAAAGTCCC |
| *BjuTT8* | STT8-1 | AAGTTACGGCTGAAGAGG | GAATCTCAACGGTCCAAT |
|  | STT8-2 | CCGTGCTTGATGGCGTTTTGGA | TTTGACTTCGGGTGGTTGTGGA |
| *BjuTT16* | STT16-1 | CAGCGTAAGGTAACGTAATA | ATCTCGGACGGATGAGTAGC |
|  | STT16-2 | GACTTAGCCTCCATTCCTTC | CCTTTATGCTTAGATTCCCC |
| *BjuTTG1* | STTG1 | CGGAAACCGCCGTCACCTAC | GCAGAACTCGCTCGTCTTGCT |
| *BjuTTG2* | STTG2 | ATGGAKGTGAAAGAGAGTRAWAG | TCAAATGGCTTGATTAGAATGTTGT |
| *BjuTT12* | STT12 | GGAATAATCTGCCAACGAGC | ACTGCTGAAGCTACCGTGAGC |
| *BjuTT19* | STT19 | ACGTGGAGACCCATTACTTT | GTTTACATCGGTACGCATCA |
