# Supplementary material for: Genome-Wide Identification, Localization, and Expression Analysis of Proanthocyanidin-Associated Genes in Brassica
Source: Front Plant Sci. 2016 Dec 9;7:1831. doi: 10.3389/fpls.2016.01831 (PMC5145881; doi:10.3389/fpls.2016.01831)
Supplement: Table S2 — Sequences of the primer pairs used in expression analysis of proanthocyanidin-associated genes of Brassica juncea. [file Table2.DOC]

Table S2Sequences of the primer pairs used in RT-PCR analysis of proanthocyanidin-associated genes of *Brassica juncea*

| Gene | Primer name | Forward sequence (5’ → 3’) | Reverse sequence (5’→ 3’) |
| --- | --- | --- | --- |
| *BjuTT3-1* | RT-DFR | CTCACAAAGAGACCGTGTGCGTAAC | CTCCATTCACTGTCGGTTTTATCAC |
| *BjuTT18-1* | RT-TT18 | AGCTTTACAAGAAGTACAAG | AGAGTTTCAGACTCAGACTT |
| *BjuTT18-3* | RT-TT18 | TTACCAAGAAGAACAAGAAG | ACTCGAACATAGAGTTTCAC |
| *BjuTT18-2* | RT-TT18 | CCTTCATTCTACACAACATGGT | TACACATAGAGTATTATATA |
| *BjuTT18-4* | RT-TT18 | CCAGACAAAAGAAGATGGTTGAAGT | CATATATGGAGACTCAAACTTG |
| *BjuANR-1* | RT-ANR | ATATACTAAGTACAATGTTTTG | TATGTGAAAATCAAGATACTTT |
| *BjuANR-3* | RT-ANR | ATATCCTAAGTACAACGTTGTG | CATATGAAAATCAAGATTATAC |
| *BjuTT19-1* | RT-TT19 | TTAACATTAATAAAAACGTTGT | GATTGTTATAAACTCAAGCTTC |
| *BjuTT19-3* | RT-TT19 | CCCAGATTAATAAAAATACTGT | CGTAATAAACCCAAGTTAATCC |
| *BjuTT19-2* | RT-TT19 | CCCAGATTAATAAAAATACTGT | ATCATCATAAGTTTGAGCTAAG |
| *Bju Actin* | Actin | AAARATGGCYGAKGSTGAKGA | CTTAGAAGCATTTYCTGTGRA |
