# Supplementary material for: Genome-Wide Identification, Localization, and Expression Analysis of Proanthocyanidin-Associated Genes in Brassica
Source: Front Plant Sci. 2016 Dec 9;7:1831. doi: 10.3389/fpls.2016.01831 (PMC5145881; doi:10.3389/fpls.2016.01831)
Supplement: Table S3 — Global statistics of the genomic assembly of Brassica juncea. [file Table3.DOC]

|  | Scaffold | | Contig | |
| --- | --- | --- | --- | --- |
|  | Size(bp) | Number | Size(bp) | Number |
| N50 | 16,777 | 12,399 | 2,584 | 65,592 |
| N60 | 10,929 | 18,533 | 1,289 | 106,592 |
| N70 | 5,465 | 29,128 | 413 | 215,317 |
| N80 | 1,239 | 60,590 | 194 | 505,047 |
| N90 | 162 | 379,783 | 141 | 972,680 |
| Longest | 224,657 | 1 | 40,430 | 1 |
| Total | 834,756,312 | 1,057,607 | 760,487,942 | 1,652,431 |
| Length>100bp | 834,756,312 | 1,057,607 | 756,677,781 | 1,599,351 |
| Length>2kb | 648,056,305 | 48,078 | 414,008,409 | 80,416 |

Table S3 Global statistics of the genomic assembly of *Brassica juncea*
